# CALEOSIN 1 interaction with AUTOPHAGY-RELATED PROTEIN 8 facilitates lipid droplet microautophagy in seedlings

**DOI:** 10.1093/plphys/kiad471

**Published:** 2023-08-24

**Authors:** Magdalena Miklaszewska, Krzysztof Zienkiewicz, Ewa Klugier-Borowska, Marcin Rygielski, Ivo Feussner, Agnieszka Zienkiewicz

**Affiliations:** Department of Plant Physiology and Biotechnology, University of Gdańsk, Wita Stwosza 59, Gdańsk 80-308, Poland; Department for Plant Biochemistry, Albrecht-von-Haller-Institute for Plant Sciences, University of Goettingen, Justus-von-Liebig-Weg 11, Goettingen 37077, Germany; Department for Plant Biochemistry, Albrecht-von-Haller-Institute for Plant Sciences, University of Goettingen, Justus-von-Liebig-Weg 11, Goettingen 37077, Germany; Centre for Modern Interdisciplinary Technologies, Nicolaus Copernicus University in Toruń, Wileńska 4, 87-100 Toruń, Poland; Centre for Modern Interdisciplinary Technologies, Nicolaus Copernicus University in Toruń, Wileńska 4, 87-100 Toruń, Poland; Centre for Modern Interdisciplinary Technologies, Nicolaus Copernicus University in Toruń, Wileńska 4, 87-100 Toruń, Poland; Department for Plant Biochemistry, Albrecht-von-Haller-Institute for Plant Sciences, University of Goettingen, Justus-von-Liebig-Weg 11, Goettingen 37077, Germany; Service Unit for Metabolomics and Lipidomics, Goettingen Center for Molecular Biosciences (GZMB), University of Goettingen, Justus-von-Liebig-Weg 11, Goettingen 37077, Germany; Department of Plant Biochemistry, Goettingen Center for Molecular Biosciences (GZMB), University of Goettingen, Justus-von-Liebig-Weg 11, Goettingen 37077, Germany; Department for Plant Biochemistry, Albrecht-von-Haller-Institute for Plant Sciences, University of Goettingen, Justus-von-Liebig-Weg 11, Goettingen 37077, Germany; Centre for Modern Interdisciplinary Technologies, Nicolaus Copernicus University in Toruń, Wileńska 4, 87-100 Toruń, Poland

## Abstract

Lipid droplets (LDs) of seed tissues are storage organelles for triacylglycerols (TAGs) that provide the energy and carbon for seedling establishment. In the major route of LD degradation (lipolysis), TAGs are mobilized by lipases. However, LDs may also be degraded via lipophagy, a type of selective autophagy, which mediates LD delivery to vacuoles or lysosomes. The exact mechanisms of LD degradation and the mobilization of their content in plants remain unresolved. Here, we provide evidence that LDs are degraded via a process morphologically resembling microlipophagy in Arabidopsis (*Arabidopsis thaliana*) seedlings. We observed the entry and presence of LDs in the central vacuole as well as their breakdown. Moreover, we show co-localization of AUTOPHAGY-RELATED PROTEIN 8b (ATG8b) and LDs during seed germination and localization of lipidated ATG8 (ATG8–PE) to the LD fraction. We further demonstrate that structural LD proteins from the caleosin family, CALEOSIN 1 (CLO1), CALEOSIN 2 (CLO2), and CALEOSIN 3 (CLO3), interact with ATG8 proteins and possess putative ATG8-interacting motifs (AIMs). Deletion of the AIM localized directly before the proline knot disrupts the interaction of CLO1 with ATG8b, suggesting a possible role of this region in the interaction between these proteins. Collectively, we provide insights into LD degradation by microlipophagy in germinating seeds with a particular focus on the role of structural LD proteins in this process.

## Teaser

CALEOSIN 1, a lipid droplet (LD) structural protein, interacts with AUTOPHAGY-RELATED PROTEIN 8 and mediates LD degradation via microautophagy during Arabidopsis seed germination.

## Introduction

Oilseed plants accumulate lipids mainly in the form of triacylglycerols (TAGs) in specialized organelles called lipid droplets (LDs; Chapman and Ohlrogge 2012; Xu and Shanklin 2016; Miklaszewska et al. 2021). LDs consist of a neutral lipid core surrounded by a phospholipid monolayer harboring a tissue-specific set of proteins (Gidda et al. 2016; Huang 2018; Fernández-Santos et al. 2020; Scholz et al. 2022). In seeds, the major structural proteins of LDs belong to 3 protein families: oleosins, caleosins, and steroleosins (Huang 2018). Besides serving as a mere carbon and energy storage depot, LDs are actually dynamic structures involved in a plethora of developmental and physiological processes in plants, such as seedling development, stress response, and pathogen resistance (Kelly et al. 2011; Blée et al. 2014; Fernández-Santos et al. 2020; Lu et al. 2020). LD dynamics and function rely on the strictly controlled balance between their efficient synthesis and degradation in cells (Zienkiewicz and Zienkiewicz 2020). During seed germination, the major route of LD degradation (lipolysis) involves lipases that hydrolyze TAGs into glycerol and free fatty acids (Graham 2008). In Arabidopsis (*Arabidopsis thaliana*), SUGAR DEPENDENT 1 (SDP1) has been shown to be responsible for breakdown of the majority of TAGs accumulated in the seeds (Eastmond 2006; Kelly et al. 2011).

Recently, a prominent role of autophagy in lipid degradation has been described in eukaryotic cells (Seo et al. 2017; Fan et al. 2019). Autophagy is a conserved catabolic process, in which cellular components, including proteins, metabolites, and entire organelles, are digested within vacuoles (yeast, plants) or lysosomes (mammals; Marshall and Vierstra 2018; Allen and Baehrecke 2020). A number of AUTOPHAGY-RELATED (ATG) proteins are directly involved in this process as core components of the cellular autophagic machinery. Among ATG proteins, ATG8 plays a prominent role in the autophagosome formation and serves as a docking site for cargos targeted for degradation by autophagy (Kellner et al. 2017; Bu et al. 2020). During this process, ATG8 proteins are lipidated by a phosphatidylethanolamine (PE) molecule via a ubiquitin-like conjugation system and anchored in the inner and outer membrane of the autophagosome (Li and Vierstra 2012; Johansen and Lamark 2020). The interaction between cargos targeted for autophagy-mediated degradation and the PE-conjugated ATG8 protein is mediated by the specific ATG8-interacting motifs (AIMs) present in autophagic receptors. Two types of ATG8-binding sites have been identified so far—AIMs in plants and yeast/LC3-interacting regions in animals (AIMs/LIRs) and ubiquitin interacting motifs (UIMs; Marshall et al. 2019). These motifs mediate the interaction of cargos with the LIR/AIM-docking site (LDS) and the UIM-docking site of ATG8, respectively. AIM/LIR comprises 4 amino acids: an aromatic amino acid (F/W/Y), followed by 2 random amino acids (X-X) and a branched-chain amino acid (L/I/V; Kellner et al. 2017; Johansen and Lamark 2020). For UIMs, no consensus amino acid sequence has been identified yet due to a limited number of known ATG8-binding UIMs across species (Marshall et al. 2019).

Two general types of autophagy have been described—macroautophagy and microautophagy (Reggiori and Klionsky 2013; Parzych and Klionsky 2014; Wang et al. 2018). In the case of macroautophagy, cellular cargo (organelles or macromolecules) is encapsulated in the cytosol by double-membrane autophagosomes, which finally fuse with lysosomes/vacuoles for their cargo delivery and degradation. In microautophagy, on the other hand, cellular components are directly engulfed by lysosomes/vacuoles and then degraded (Parzych and Klionsky 2014; Schuck 2020). A type of selective autophagy directly involved in LD degradation has been termed lipophagy. In yeast, microlipophagy plays a predominant role in this process via ATG-dependent or ATG-independent mechanisms (van Zutphen et al. 2014; Vevea et al. 2015; Seo et al. 2017). In mammalian cells, in addition to microlipophagy, a complete or “piecemeal” macrolipophagy is involved in LD turnover through de novo autophagosome formation, followed by autophagic cargo degradation in the autolysosome (Ward et al. 2016; Khawar et al. 2021).

The most recent studies also suggest an important role of lipophagy in LD breakdown in algae and plants (Fan et al. 2019; Barros et al. 2020; Zienkiewicz et al. 2020a). Microlipophagy-like degradation of LDs has been described for various microalgae like *Chlamydomonas reinhardtii*, *Auxenochlorella protothecoides*, or *Nannochloropsis oceanica* (Zhao et al. 2014; Tsai et al. 2018; Zienkiewicz et al. 2020a). In land plants, autophagy seems to be involved in LD degradation during anther development and pollen germination (Kurusu et al. 2014; Zhao et al. 2020). A microlipophagy-resembling process has also been described during dark-induced starvation in Arabidopsis leaves (Fan et al. 2019). This process was impaired in Arabidopsis *atg2-1 sdp1-4* and *atg5-1 sdp1-4*, suggesting a key role of core macroautophagy machinery components in degradation of LDs. The contribution of autophagy to LD degradation has also been suggested during seed germination in Arabidopsis in an LD structural protein mutant—*CALEOSIN 1* (*CLO1*; Poxleitner et al. 2006). Loss of function of CLO1 was associated with delayed LD mobilization when compared with wild-type plants. Interestingly, unlike the wild-type plants, *clo1* mutants showed the absence of LDs inside the vacuoles in cotyledon cells. Thus, it has been suggested that CLO1 may be directly involved in microlipophagy during Arabidopsis seed germination (Poxleitner et al. 2006).

Deciphering the molecular mechanisms behind LD degradation via lipophagy is still in its infancy; however, recent findings suggest the direct interaction of LD structural proteins with the autophagic machinery (Marshall et al. 2019; Zienkiewicz et al. 2020a). Our previous studies showed that the LIPID DROPLET SURFACE PROTEIN from *N. oceanica* interacts with *No*ATG8 via its AIM (Zienkiewicz et al. 2020a). Similarly, Marshall et al. (2019) showed that Arabidopsis OLEOSIN 1 (OLE1) binds to the LDS of ATG8e. The fact that structural LD proteins are able to interact with ATG8, together with the observation that *clo1* mutants of Arabidopsis are impaired in the degradation of LDs inside the vacuoles (Poxleitner et al. 2006), prompted us to investigate whether caleosins mediate LD degradation by interaction with ATG8 and to decipher the mechanism of this process. To date, only a few of the 8 caleosins (CLO1 to CLO8) identified in the Arabidopsis genome have been characterized (Næsted et al. 2000; Shen et al. 2014). All caleosins contain an N-terminal hydrophilic domain consisting of an EF-hand calcium-binding motif, a central hydrophobic region with the proline knot that anchors the protein to the LD membrane, and a C-terminal hydrophilic region with several phosphorylation- and heme-binding sites (Purkrtova et al. 2007; Chapman et al. 2012; Huang 2018). The genes encoding CLO1 and CLO2 are highly expressed in developing seeds, suggesting their role in seed germination or dormancy (Poxleitner et al. 2006; Liu et al. 2022). Consequently, here we examine in detail the phenotypic effects of *CLO1* and *CLO2* mutations on LD degradation during Arabidopsis seed germination and provide evidence for the interaction of the selected caleosins with ATG8. Our study also demonstrates the possible role of CLO1 in LD degradation by facilitating microlipophagy during seed germination.

## Results

### Characterization of *clo1* and *clo2* mutants

The genome of Arabidopsis contains 8 caleosin genes (Shen et al. 2014). Two of them—*CLO1* (At4g26740) and *CLO2* (At5g55240) are expressed preferentially in developing seeds (Shen et al. 2014). To determine the function of Arabidopsis CLO1 and CLO2 in seeds, we characterized T-DNA insertion lines available for *CLO1* (*clo1-1*; Poxleitner et al. 2006) and *CLO2* (SALK_046559) in the Columbia 0 (Col-0) ecotype. PCR genotyping was performed to identify *clo1* and *clo2* homozygous mutant lines (Supplemental Fig. S1, A and B). Additionally, immunoblotting analyses showed that both *clo1* and *clo1 clo2* mutants failed to accumulate CLO1 protein in the mature seeds (Supplemental Fig. S1C). To investigate the effects of the loss of function of CLO1 and CLO2 on seed morphology, we evaluated 3 different traits for seed yield in *clo1*, *clo2,* and *clo1 clo2* mutants, seed dry weight, seed length, and seed width (Supplemental Fig. S2), and compared them with wild-type plants (Col-0). The mature dry seeds obtained from the *clo1 clo2* double mutant were significantly heavier when compared with both Col-0 and *clo1* mutants, and they were also longer than *clo1* (Supplemental Fig. S2, B and C). No significant differences were observed in seed width between the analyzed lines (Supplemental Fig. S2D). No significant changes in seed morphometry were observed between the single mutants and the control plants (Supplemental Fig. S2, A to D).

### Disruption of CLO1- and CLO2-encoding genes affects acyl composition of mature and germinating seeds of Arabidopsis

**Figure 1.**
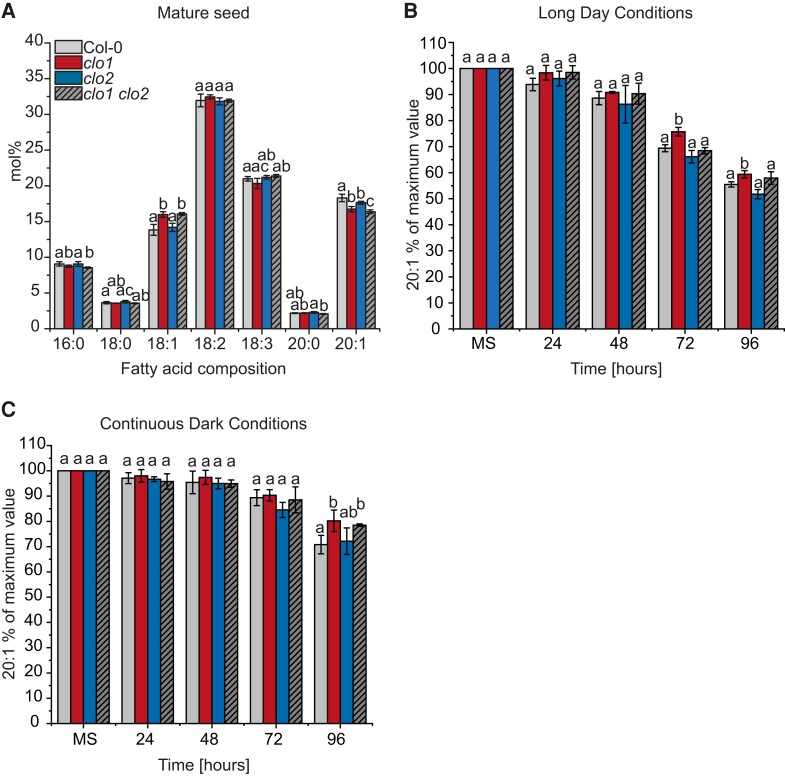
Mutation in *CLO1*, but not in *CLO2,* affects TAG degradation. **A)** Changes in mature seed (MS) FA composition between Col-0, *clo1*, *clo2*, and *clo1 clo2*. **B** and **C)** Changes in 20:1 content are presented as a percentage of the amount determined in the same number of mature seeds. The analysis was performed during germination course under long day **B)** and continuous dark **C)** conditions. Data are means ± Sd from 2 independent experiments with 6 biological replicates (*n* = 6). The experiment was repeated 3 times with similar results using independent biological samples. Statistical analysis was performed by 1-way ANOVA with Tukey’s post hoc test. Different letters indicate significant differences with *P* < 0.05. 16:0, palmitic acid; 18:0, stearic acid; 18:1, oleic acid; 18:2, linoleic acid; 18:3, linolenic acid; 20:0, arachidic acid; 20:1, eicosenoic acid; 24, 48, 72 and 96 h—time of seed germination.

To address the role of CLO1 and CLO2 in lipid metabolism, we first analyzed the acyl composition of total lipids from mature and germinating seeds of single and double mutants of *CLO1* and *CLO2* under diverse light conditions and compared them with the control plants (Fig. 1, Supplemental Figs. S3 and S4). In the mature seeds, the mutation in the CLO1-encoding gene was accompanied by a significant increase in 18:1 content and lower levels of 16:0 compared with Col-0 (Fig. 1A). Moreover, all analyzed *clo1* and *clo2* mutants showed a significant reduction in 20:1 compared with Col-0. During the course of germination of all analyzed mutants under long day conditions, a similar pattern of 18:1 and 20:1 content was observed, with exception to 96 h of germination, where no significant changes of the acyl content were found among *clo1*, *clo2*, and *clo1 clo2* seedlings in comparison with Col-0 plants (Supplemental Fig. S3). Similar changes in 18:1 and 20:1 levels were observed during in vitro germination under continuous dark conditions (Supplemental Fig. S4). As 20:1 is found almost exclusively in TAGs in Arabidopsis seeds, it has been commonly used as a marker of TAG degradation (Kunst et al. 1992; Poxleitner et al. 2006; Kelly et al. 2011). Consequently, to determine whether the *clo1* and *clo2* mutants are impaired in TAG breakdown, we analyzed the changes in their 20:1 content during in vitro seed germination under long day (Fig. 1B) and continuous dark conditions (Fig. 1C). In both cases, degradation of 20:1 was observed for all analyzed plants; however, degradation was less obvious for seeds germinating under continuous dark conditions. Under long day conditions, the content of 20:1 was significantly higher in *clo1* plants after 72 and 96 h of germination, when compared with *clo2* and Col-0 (Fig. 1B). In contrast, under continuous dark conditions, the content of 20:1 was significantly higher for both *clo1* and *clo1 clo2* seeds compared with Col-0 but only after 96 h of germination (Fig. 1C). Overall, the breakdown of 20:1 was slower during in vitro germination in the dark, independent from the *CLO1* and *CLO2* mutations. However, mutation in *CLO1* resulted in a slight delay of this process regardless of the light conditions.

### Cellular organization of *clo1* mutant reveals disrupted LD breakdown

**Figure 2.**
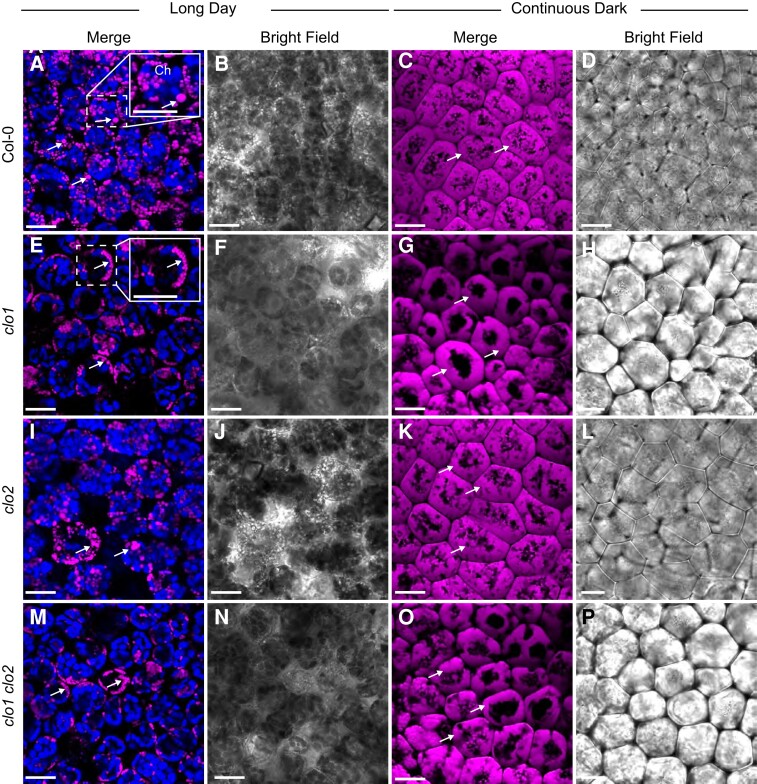
LDs accumulate around the central vacuole in *clo1* and *clo1clo2*. Representative CLSM images of the cotyledon cells of Col-0 (**A** to **D**), *clo1* (**F** to **H**), *clo2* (**I** to **L**), *clo1 clo2* (**M** to **P**) after 48 h of germination under long day and continuous dark conditions showing LDs stained with BODIPY 505/515 (arrows) and chlorophyll (Ch). The insets in **A** and **E** correspond to magnified areas marked with the dashed line. Bars = 15 *µ*m.

**Figure 3.**
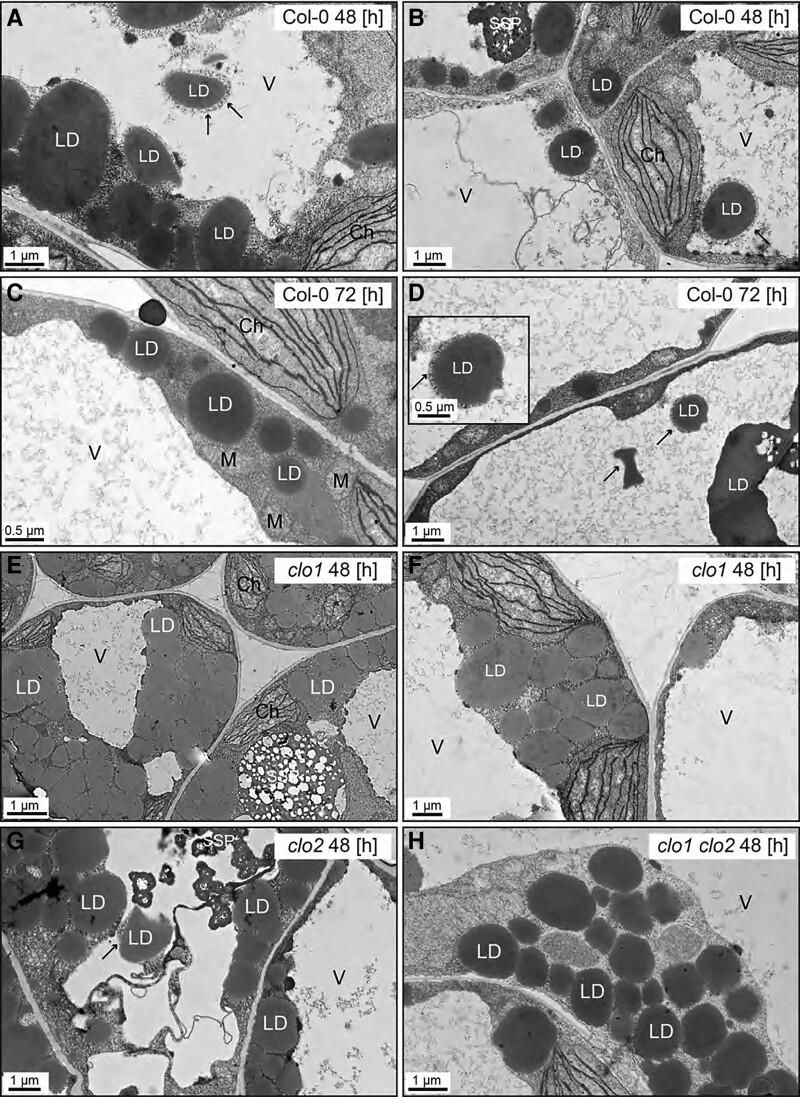
Disruption of *CLO1*, but not of *CLO2*, impairs LDs entering the central vacuole. Representative TEM images of Arabidopsis cotyledon cells. **A–D**) Ultrastructure of Col-0 cotyledon cells after 48 h (**A** and **B**) and 72 h (**C** and **D**) of in vitro germination. LDs are present in the cytoplasm as well as inside the vacuole. The LDs visible in the area of the vacuole are surrounded by cytoplasmic material (arrows). (**E** and **F**) Ultrastructure of *clo1* cotyledon cells after 48 h of in vitro germination. LDs are localized only in the cytoplasm around the vacuole. **G)** Ultrastructure of *clo2* cotyledon cells after 48 h of in vitro germination. LDs can be seen in the cytoplasm and inside the vacuole. **H)** Ultrastructure of *clo1 clo2* cotyledon cells after 48 h of in vitro germination. LDs occupy only the cytoplasmic area. Ch, chloroplast; LD, lipid droplet; M, mitochondrion; SSP, seed storage protein; V, vacuole.

To confirm the impact of *CLO1* and *CLO2* mutations on TAG degradation during seed germination, we analyzed the cellular organization of LDs in cotyledons of *clo1*, *clo2*, and *clo1 clo2* seedlings with reference to Col-0 using confocal laser scanning microscopy (CLSM) and transmission electron microscopy (TEM; Figs. 2 and3). At 48 h of in vitro germination under long day conditions, in both Col-0 and *clo2* cotyledons, LDs were found to be randomly distributed throughout the cells between the forming chloroplasts (Fig. 2, A, B and I, J, arrows). In contrast, in the cells of *clo1* and *clo1 clo2* mutants, we found that LDs were usually present in the peripheral parts of the cells and were often accumulated together, forming clumps (Fig. 2, E, F and M, N, arrows). As expected, under continuous dark conditions, in all the analyzed lines, no chloroplasts could be found at 48 h of germination. However, similar to long day conditions, evident changes in LD behavior were found for single and double *clo1* mutants when compared with Col-0 and *clo2*. In the latter case, numerous LDs localized at the periphery and in the center of the cells (Fig. 2, C, D and K, L, arrows), whereas in the vast majority of *clo1* and *clo1 clo2* cotyledon cells, LDs were mostly concentrated in the peripheral area of the cell around the central vacuole (Fig. 2, G, H and O, P, arrows). To gain more detailed insights into the cellular effects of *CLO1* and *CLO2* disruption in seeds germinating under long day conditions, we analyzed the ultrastructure of *clo1*, *clo2*, and *clo1 clo2* cotyledon cells with reference to Col-0 (Fig. 3). After 48 h of germination, in the cotyledon cells of Col-0, the most prominent feature was the presence of LDs at the border as well as inside the central vacuole (Fig. 3, A to D). Moreover, these LDs were usually surrounded by a thin layer of cytoplasmic material (Fig. 3, A and B, arrows). At the later steps of germination, in most of the cells, partially degraded LDs and residues of lipidic material were found in the central vacuole (Fig. 3D). At the same time, the cytosolic pool of LDs was strongly depleted and composed mostly of small LDs (Fig. 3, C and D). Strikingly, at 48 h of *clo1* germination, practically no LDs were observed in the central vacuole (Fig. 3, E and F). Instead, they were found as tightly packed structures in the cytosol, forming aggregates and pushing toward the tonoplast. In the case of *clo2* plants, there were no obvious differences in the ultrastructure compared with Col-0, as the LDs were present inside the central vacuole and were also carrying cytosolic material on their surface (Fig. 3G, arrow). Finally, the double mutation of *CLO1* and *CLO2*, similarly to the *clo1* ultrastructural phenotype, was accompanied by the presence of numerous large LDs in the cytoplasm (Fig. 3H). No LDs were observed inside the central vacuole of *clo1 clo2* cotyledons during the whole course of germination. These results show that the disruption of *CLO1*, but not of *CLO2*, is associated with the absence of LDs in the central vacuole during Arabidopsis seed germination.

### In wild-type Arabidopsis, the vacuolar breakdown of LDs occurs in a process that resembles microlipophagy

**Figure 4.**
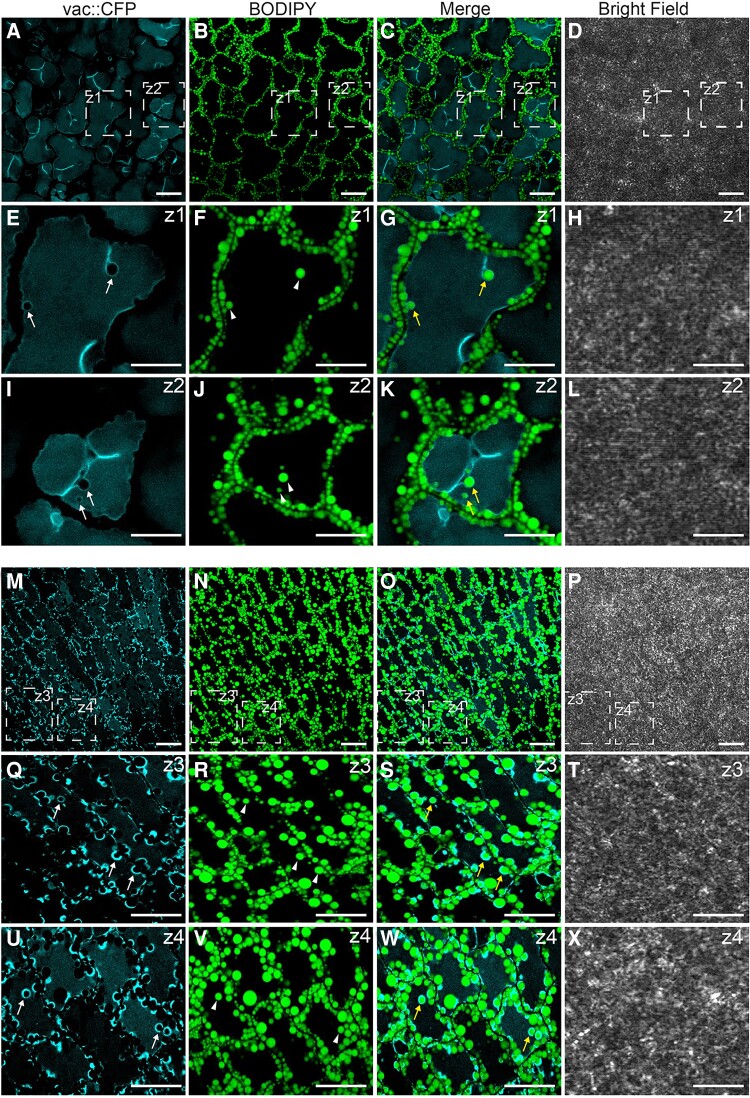
Subset of LDs is degraded via microlipophagy during seed germination. Representative CLSM images of the cotyledon cells of Col-0 after 36 h **A to L)** and 48 h **M–X)** of seed germination under long day conditions. LDs were stained with BODIPY 505/515. Tonoplast marker protein fused to the CFP is visible as blue. The white arrows indicate tonoplast invaginations, the arrowheads indicate LDs, and the yellow arrows indicate LDs surrounded by CFP-labeled tonoplast invaginations. The areas marked with dashed lines (**Z**) represent zoomed views of the corresponding image. Bars = 10 *µ*m.

**Figure 5.**
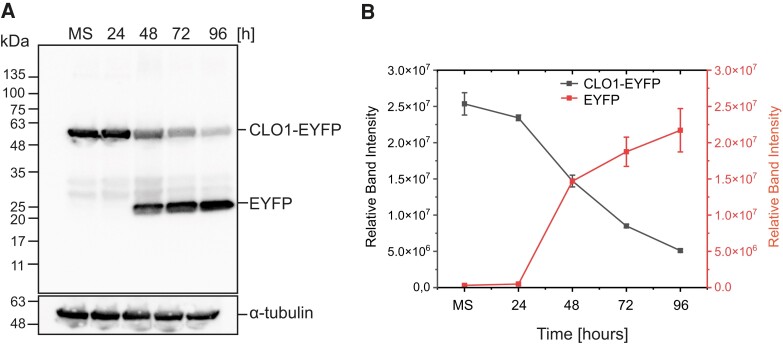
Accumulation of free EYFP derived from CLO1-EYFP occurs during seed germination. **A)** Immunoblot analysis of CLO1-EYFP and free EYFP pools during seed in vitro germination; α-tubulin was used as a loading control. **B)** Densitometric data corresponding to CLO1-EYFP and free EYFP bands from **A)**. Each data point represents the average of 3 independent experiments. Values represent the means ± Sd (*n* = 3). CLO1, CALEOSIN 1; EYFP, enhanced yellow fluorescent protein; MS, mature seed; 24, 48, 72 and 96 h—time of seed germination.

**Figure 6.**
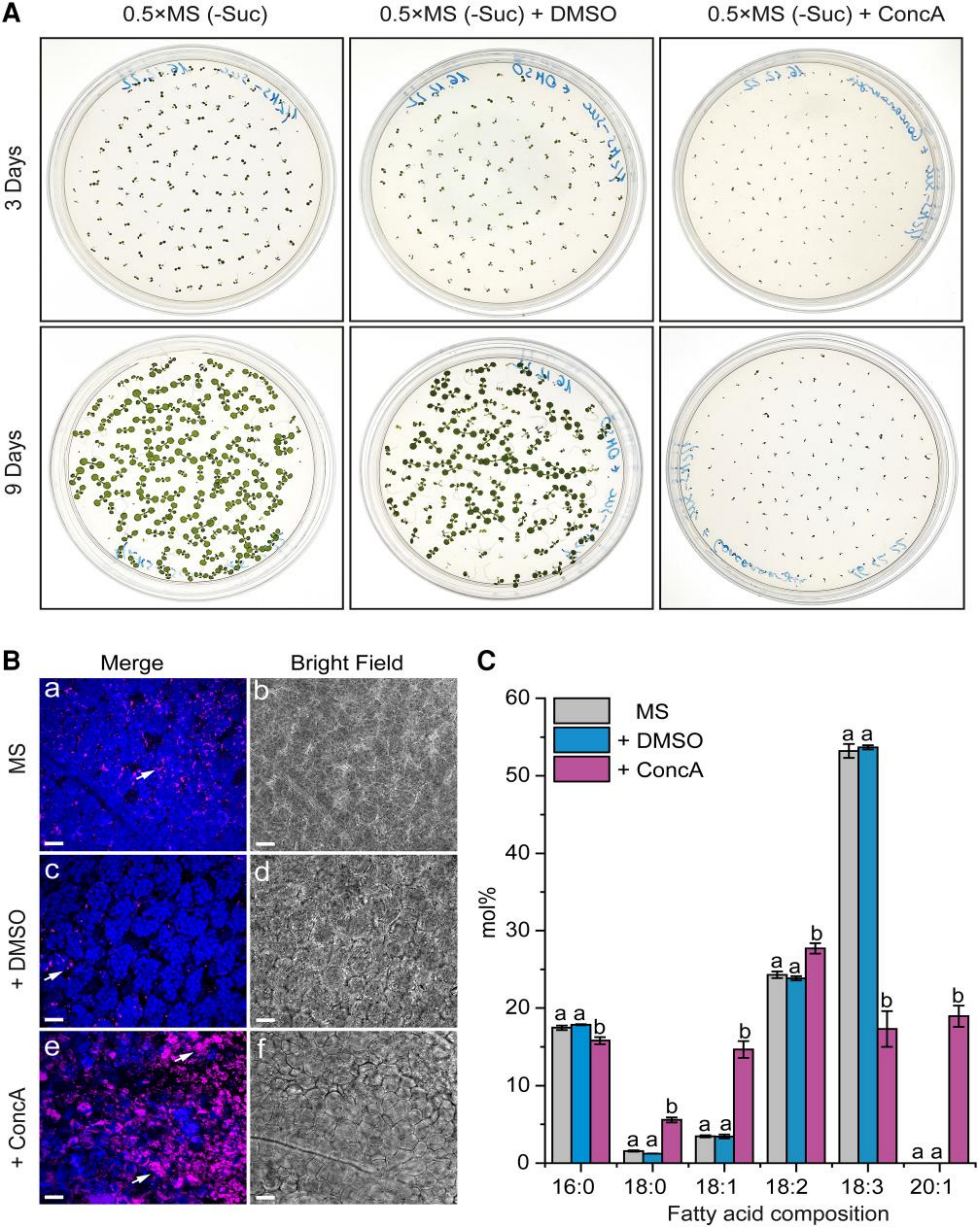
ConcA treatment hampers seed germination and TAG degradation. **A)** Comparison of seed germination and seedling growth after 3 d (upper panel) and 9 d (lower panel) of cultures growing on control (0.5×MS-Suc), solvent control (DMSO), and ConcA-containing media. **B)** Representative CLSM images of the cotyledon cells of 9-d-old seedlings from **A)**. The arrows indicate LDs, and chloroplasts are shown in blue. **C)** Comparison of lipid FA composition between 9-d-old seedling cultures from **A)**. Data are means ± Sd from 2 independent experiments with 6 biological replicates (*n* = 6). Statistical analysis was performed by 1-way ANOVA with Tukey’s post hoc test. The different letters indicate significant differences with *P* < 0.05. 0.5×MS-Suc, Murashige and Skoog (0.5×MS) medium (without sucrose; -Suc); DMSO, dimethyl sulfoxide. Bars = 15 *µ*m.

To gain more information on the cellular mechanisms of LD degradation, we labeled LDs with BODIPY 505/5015 in transgenic Arabidopsis plants overexpressing a tonoplast marker protein fused to the cyan fluorescent protein (vac::CFP). Then, we analyzed the behavior of LDs during the course of seed germination using CLSM (Fig. 4). After 36 h (Fig. 4, A to L) and 48 h (Fig. 4, M to X) of seed germination under long day conditions, we detected individual LDs (Fig. 4, arrowheads) inside the vacuoles. These LDs were often associated with invaginations (36 h) or directly surrounded (48 h) by the CFP-labeled tonoplast (Fig. 4, yellow arrows). Moreover, we observed a similar localization pattern for LDs during the microscopic analysis of the plants expressing the *CLO1-EYFP* (enhanced yellow fluorescent protein) construct (Supplemental Fig. S5). In the cotyledon cells of germinating seeds, during the early stages of seed germination (24 h), the CLO1-EYFP signal was observed in the form of numerous rings located in the cytoplasm (Supplemental Fig. S5, arrowheads). However, the later steps of seed germination (48 h) were accompanied by a rich pool of these structures present in the area of the central vacuole (Supplemental Fig. S5, arrows). Moreover, using immunoblotting, we followed the levels of CLO1-EYFP and free EYFP during seed germination (Fig. 5). We found that the pool of CLO1-EYFP decreased during the time course of seed germination, with a simultaneous enrichment of the free EYFP pool. As free GFP and its derivatives are resistant to degradation inside the vacuole (Toyooka et al. 2006; Fan et al. 2019), the observed pattern suggests that CLO1-EYFP-labeled LDs undergo breakdown in the vacuole. To further confirm the relationship between autophagy and LD degradation, we used an autophagy inhibitor concanamycin A (ConcA). The treatment with ConcA severely inhibited seed germination and postgerminative growth (Fig. 6A). Moreover, we found that inhibition of autophagy with ConcA strongly hampers LD breakdown (Fig. 6B) and TAG degradation (Fig. 6C). Unlike in the control plants, in the cotyledon cells of 9-d-old seedlings treated with ConcA, we observed a strong accumulation of LDs (Fig. 6B) and high levels of 20:1 (Fig. 6C), respectively. Taken together, our observations strongly suggest that degradation of a subset of LDs during seed germination is mediated by microlipophagy.

### ATG8 protein is associated with LDs during seed germination

**Figure 7.**
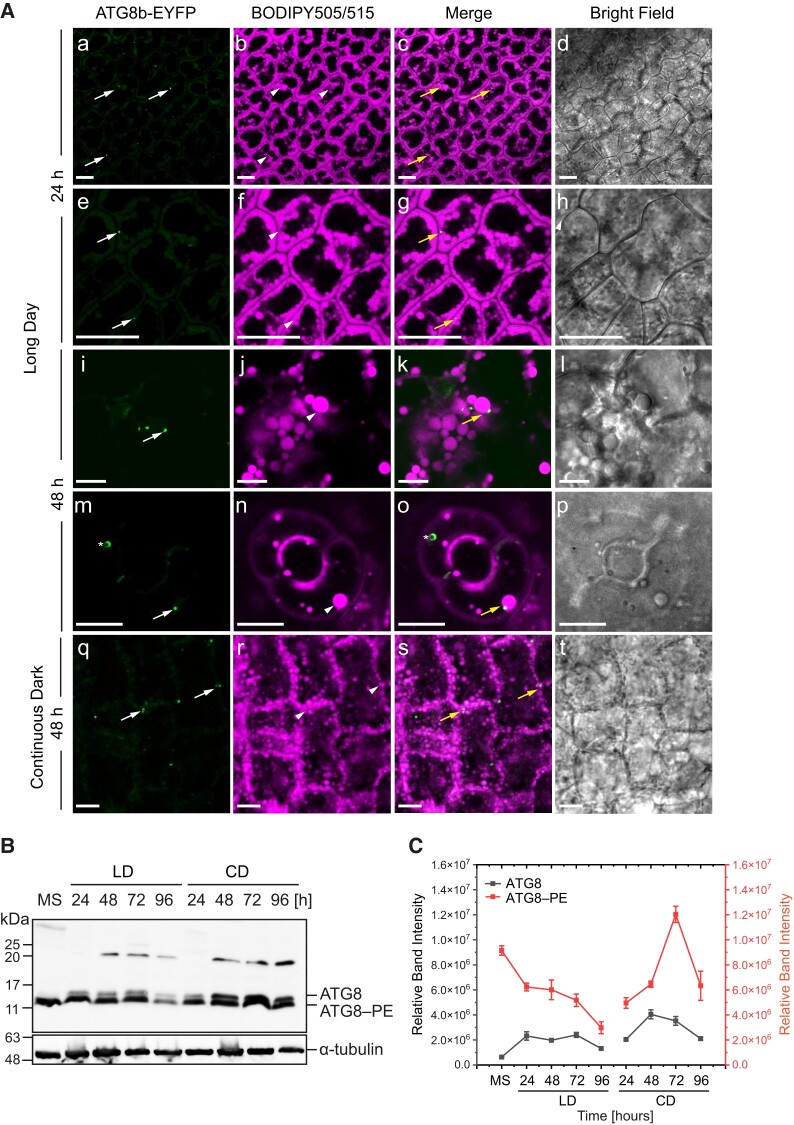
ATG8b and LDs co-localize in cotyledon cells. **A)** Representative CLSM images of the cotyledon cells of ATG8b-EYFP transgenic line (green) stained with BODIPY 505/515 (magenta) and analyzed after 24 h (a to h) and 48 h (i to p) of seed germination under long day conditions as well as after 48 h (q to t) under continuous dark conditions. The white arrows indicate ATG8b-EYFP, the arrowheads indicate LDs, and the yellow arrows indicate co-localization of both. The asterisk (m to o) indicates autophagosome labeled with ATG8b-EYFP. Bars = 10 *µ*m. **B)** Immunoblot analysis of ATG8 and ATG8–PE abundance during seed in vitro germination under long day (LD) and continuous dark conditions; α-tubulin was used as a loading control. **C)** Densitometric data corresponding to ATG8 and ATG8–PE bands from **B)**. Each data point represents the average of 3 independent experiments. Values represent the means ± **Sd** (*n* = 3). EYFP, enhanced yellow fluorescent protein; MS, mature seed; 24, 48, 72 and 96 h—time of seed germination.

To further examine whether autophagy plays an essential role in LD degradation, we analyzed a possible interaction between ATG8b and LDs (Fig. 7A). We confirmed in situ co-localization of ATG8b with LDs in the cotyledon cells (Fig. 7A). Using plants stably expressing the *ATG8b-EYFP* construct (Supplemental Fig. S6), we demonstrated that in cotyledons ATG8b co-localizes with LDs during seed germination under both long day (Fig. 7A, a to p, yellow arrows) and continuous dark conditions (Fig. 7A, q to t, yellow arrows). This co-localization was observed in the mesophyll cells as well as in the stomata (Fig. 7A, m to p). Additionally, using an anti-ATG8 antibody, we were able to detect 2 pools of ATG8 protein, free ATG8 and ATG8–PE, in Col-0 seeds germinating in vitro under long day and continuous dark conditions (Fig. 7B). Under long day conditions, ATG8 abundance increased just after the start of seed germination and remained stable until the last analyzed time point (96 h), when a slight decrease was observed. We observed a similar pattern for ATG8 under continuous dark conditions (Fig. 7, B and C). The levels of ATG8–PE were found to be higher than ATG8 under both conditions. Moreover, seed germination under continuous dark conditions was accompanied by a stronger accumulation of ATG8–PE, when compared with long day conditions.

To examine whether CLO1 and ATG8 are indeed associated with LDs during seed germination, we employed the 2 above-mentioned antibodies to determine the presence of both proteins in the protein extracts isolated from LD fractions obtained after 24 h of germination from Col-0, *clo1*, *clo2*, and *clo1 clo2* seeds (Supplemental Fig. S7, A to C). As expected, specific protein bands corresponding to CLO1 were detected in LD fractions isolated from Col-0 and *clo2* seeds but not in those extracted from *clo1* and *clo1 clo2* seeds (Supplemental Fig. S7A). When the anti-ATG8 antibody was used, the most prominent band observed in the LD fractions extracted from all analyzed plant lines corresponded to ATG8–PE, whereas ATG8 was detected at a much lower level (Supplemental Fig. S7B). The findings from these experiments suggest that mostly ATG8–PE is associated with LDs and that this association is unaffected by *CLO1* and *CLO2* mutations.

### Arabidopsis caleosins contain putative AIMs and interact with ATG8 proteins

**Figure 8.**
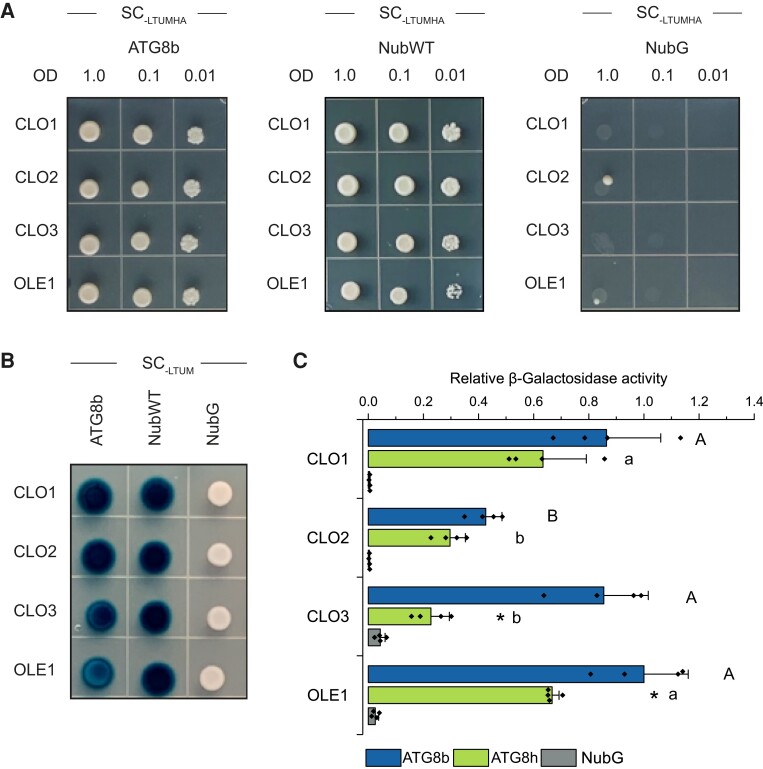
Caleosins interact with ATG8 proteins. **A)** Yeast mating–based split-ubiquitin assay for interaction of the Cub fusions of CLO1, CLO2, CLO3, or OLE1 (bait) with the NubG fusion of ATG8b (prey). Diploid yeasts carrying the given bait and prey constructs were spotted on the SC medium without Leu, Trp, Ura, Met, His, and Ade (SC-LTUMHA). Serial dilutions of yeast culture are as indicated. **B)** X-Gal overlay assay for detection of β-galactosidase activity for the same bait and prey combinations as in **A)**. Diploid yeasts were spotted on the SC medium without Leu, Trp, Ura, and Met (SC-LTUM). For **A)** and **B)**, NubG and NubI (NubWT) served as a negative and a positive control, respectively. As an additional positive control, interaction between OLE1 and ATG8 proteins was used. **C)** Quantitative β-galactosidase activity assay for the Cub fusions of CLO1, CLO2, CLO3, or OLE1 (bait) with the NubG fusions of ATG8b or ATG8h (prey). NubG was used as a negative control. Data are means ± Sd from 4 independent yeast transformants. The β-galactosidase activity was normalized relative to the activity measured for the interaction between OLE1 and ATG8b. Statistical analysis was performed by 1-way ANOVA with Tukey’s post hoc test and an unpaired 2-tailed Student’s *t*-test. The different letters indicate significant (Tukey’s test, *P* < 0.01) differences between baits’ interactions with ATG8b (uppercase) or ATG8h (lowercase). An asterisk denotes significant differences (unpaired 2-tailed Student’s *t*-test, *P* < 0.01) between interaction with ATG8b and interaction with ATG8h for the same bait. SC, synthetic complete medium.

ATG8 interactors typically possess the AIM/LIR motif with the consensus sequence W/F/Y-X-X-L/I/V (Noda et al. 2010; Liu et al. 2021). As caleosins may potentially mediate LD degradation via autophagy, we were interested if they possess putative AIMs. Therefore, by using the iLIR software (Kalvari et al. 2014), we analyzed the amino acid sequences of the 8 Arabidopsis caleosins and found that each of them contains at least 1 putative AIM (Supplemental Table S1 and Fig. S8). The AIM1 (YXXL), localized just before the proline knot, was identified only in CLO1, CLO2, CLO3, and CLO8, whereas the AIM2 (WXXL) was found at the C-termini of all analyzed caleosins, except CLO8, which is truncated compared with the others. To verify whether caleosins directly bind to ATG8, we first examined their interaction using the mating-based split-ubiquitin yeast system. In this approach, we analyzed the ATG8-binding properties of CLO1, CLO2, and CLO3 as well as OLE1, as a positive control. NubWT was used as another positive control to demonstrate that Cub fusion constructs are properly expressed in the yeast cells (Supplemental Fig. S9C). As shown in Fig. 8, A and B, all tested proteins interacted with ATG8b. A quantitative β-galactosidase assay also revealed that the interaction with ATG8b was comparable for CLO1, CLO3, and OLE1, whereas 2-fold weaker β-galactosidase activity was found for CLO2 (Fig. 8C). When ATG8h was used as a prey, detected β-galactosidase activities were lower compared with ATG8b (Fig. 8C).

**Figure 9.**
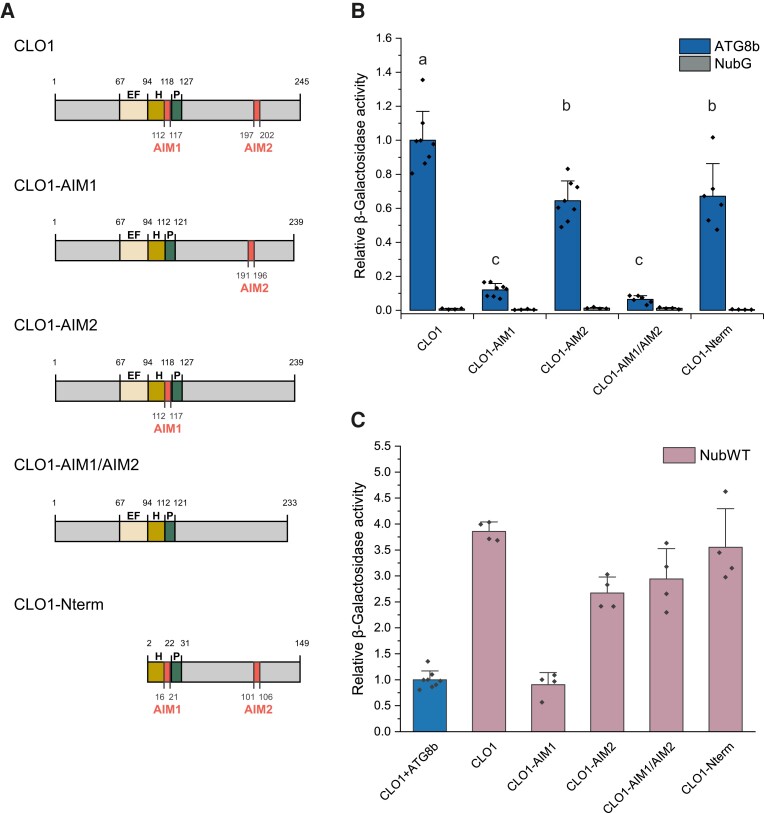
Deletion of AIM1 leads to disruption of the interaction between CLO1 and ATG8. **A)** Schematic diagram of tested CLO1 variants, lacking AIM1 (112 to 117 aa; CLO1-AIM1), AIM2 (196 to 202 aa; CLO1-AIM2), both AIM1 and AIM2 (CLO1-AIM1/AIM2) and N-terminus (1 to 97 aa, including EF-domain). The EF-hand domain, the central helix, and the proline knot are denoted as EF, H, and P, respectively. **B)** Quantitative β-galactosidase activity assay Cub fusions of CLO1 variants shown in **A)** (bait) with the NubG fusion of ATG8b (prey). NubG was used as a negative control. Data are means ± Sd from 6 to 8 independent yeast transformants. The β-galactosidase activity was normalized relative to the activity measured for the interaction between CLO1 and ATG8b. Statistical analysis was performed by 1-way ANOVA with Tukey’s post hoc test. The different letters indicate significant (*P* < 0.01) differences between β-galactosidase activity for the tested variants. **C)** Quantitative β-galactosidase activity assay for the Cub fusions of CLO1 variants shown in **A)** with NubWT (positive control). Data are means ± Sd from 8 (CLO1) or 4 (CLO1 variants) independent yeast transformants. The β-galactosidase activity was normalized relative to the activity measured for the interaction between CLO1 and ATG8b (blue bar).

To test whether AIMs are required for interaction of CLO1 with ATG8b, we generated several CLO1 AIM mutants: CLO1-AIM1 (112 to 117 aa) with deletion of the entire AIM1, CLO1-AIM2 (196 to 202 aa) with deletion of the entire AIM2, and CLO1-AIM1/AIM2 with deletion of both AIM1 and AIM2 (Fig. 9A). For this step, we had selected the AIMs with the highest position-specific scoring matrix (PSSM) score (AIM2, Supplemental Table S1), excluding the ones with a PSSM score below 8 (Supplemental Table S2), except the AIM corresponding to xLIR in CLO3 (AIM1). Additionally, we generated a CLO1 mutant with the deleted N-terminus (1-97 aa, including EF-domain; Fig. 9A). We found that CLO1-AIM1 and CLO1-AIM1/AIM2 significantly lost their ability to interact with ATG8b, as we observed a 90% decrease in β-galactosidase activity (Fig. 9B, Supplemental Fig. S9A). A significant but less prominent decrease in the binding efficiency to ATG8b was also observed for CLO1-AIM2. Similar results were obtained for the interaction of CLO1 mutants with ATG8h (Supplemental Fig. S9B). The proper expression of Cub fusion constructs was assessed by measuring the interaction with NubWT (Fig 9C).

As the secondary approach to confirm the binding of CLO1 to ATG8, we performed co-immunoprecipitation assays using the anti-CLO1 antibody. We found that ATG8–PE co-immunoprecipitated with CLO1 from the LD protein extracts from imbibed seeds of Col-0 but not of *clo1* and *clo1 clo2* mutants (Supplemental Fig. S10). Together, these data suggest that seed caleosins, CLO1 and CLO2 interact with ATG8 proteins and that the putative AIMs identified in CLO1 may be involved in this interaction.

## Discussion

In this study, we showed that autophagy is involved in LD degradation during Arabidopsis seed germination. Our results demonstrate that lipophagy occurs in a CLO1-dependent process that resembles microlipophagy. Moreover, we provided evidence that autophagic marker protein ATG8 co-localizes with LDs in Arabidopsis seedlings and interacts with LD structural proteins, such as CLO1, CLO2, CLO3, and OLE1, in the yeast system. Consequently, we report here additional insights into LD degradation during Arabidopsis seed germination.

### A process resembling microlipophagy is involved in LD breakdown

Our observations of Col-0 germinating seeds overexpressing the tonoplast marker protein fused to CFP and labeled for LDs showed that individual LDs found in vacuoles often associate with the tonoplast invaginations. We also observed accumulation of free EYFP derived from CLO1-EYFP-labeled LDs during seed germination, which suggests degradation of LDs within the vacuole. Moreover, the treatment with ConcA, commonly used as an autophagy inhibitor (Yano et al. 2015; Couso et al. 2018), resulted in hampering TAG degradation and accumulation of LDs in the cotyledon cells. To date, most reports have described the role of microlipophagy in LD degradation in animal, yeast, algae, and plant nonseed tissues (for review, see Zienkiewicz and Zienkiewicz 2020; Xu and Fan 2022). In the model green microalga *C. reinhardtii*, microlipophagy was detected under nitrogen resupply (NR) conditions, where LDs were present and degraded in the vacuolar lumen (Tsai et al. 2018). Similarly, microlipophagy-like degradation of LDs was observed in *N. oceanica* cells under NR conditions (Zienkiewicz et al. 2020a). LD breakdown by microlipophagy has been also recently demonstrated in developing and germinating pollen of angiosperms (Zhao et al. 2020; Akita et al. 2021). Fan et al. (2019) showed that LDs found inside the vacuole or within invaginations of the tonoplast in dark-treated Arabidopsis rosette leaves undergo degradation via a microlipophagy-resembling process. Overall, our findings show that in germinating seeds a certain pool of LDs is transported to the vacuoles for their degradation by tonoplast invaginations in a process resembling microlipophagy.

### No LDs are observed in *clo1* in the vacuoles during seed germination

During germination of both Col-0 and *clo2* seeds, LDs appeared in the central vacuoles together with the cytoplasmic material, which is characteristic for microlipophagy. Consequently, the later steps of seed germination were accompanied by the presence of LDs undergoing degradation within the vacuole in these plants. In contrast, in *clo1* and *clo1 clo2* mutants, no LDs were found in the vacuoles. Instead, they accumulated in the peripheral parts of the cells, forming aggregates around the vacuoles. Similar observations have been reported previously for *clo1* plants by Poxleitner et al. (2006). These findings led the authors to hypothesize that at least a certain pool of LDs might be degraded by a caleosin-dependent mechanism, which involves microautophagy and finally leads to degradation of LDs in the vacuoles (Poxleitner et al. 2006). We observed such a similar distribution of LDs, which was particularly visible during germination of *clo1* and *clo1 clo2* seeds under continuous dark conditions. Our results thus support the hypothesis of Poxleitner and colleagues that the loss of function of CLO1 affects the transfer of LDs to the vacuoles and in consequence their degradation via the microlipophagy pathway. Here, we further show that the disruption of another seed caleosin, CLO2, does not have this effect.

### Loss of function of CLO1 affects seed lipid composition and TAG degradation rates

Disruption of *CLO1* had a detectable impact on the acyl composition of total lipids in Arabidopsis, resulting in the increase of 18:1 and lower levels of 20:1 in mature seeds. The 20:1 level was also significantly lower in *clo2* seeds compared with Col-0. Similar changes in lipid composition in *clo1* and *clo2* were reported recently by Liu et al. (2022) and linked to the role of both caleosins in lipid biosynthesis during Arabidopsis seed development (Liu et al. 2022). Moreover, in silico analysis of Arabidopsis caleosins showed the presence of RY and RAV1 motifs in their promoters which can bind transcription factors regulating oil synthesis during seed development (Shen et al. 2014). Interestingly, suppression of the *OLE1* gene in Arabidopsis had an opposite effect on 18:1 and 20:1 content in mature seeds (Siloto et al. 2006). These findings suggest that core proteins of LDs, besides their structural function, may also regulate acyl composition of TAGs that accumulate in LDs during embryo development. Moreover, previous studies on *clo1* mutants demonstrated slower degradation of TAGs in seeds of the *clo1* mutant between 48 and 60 h of germination, which was identified by elevated levels of 20:1 when compared with wild-type seedlings (Poxleitner et al. 2006). We confirmed such a profile of 20:1 in both *clo1* and *clo1 clo2* seeds during later steps of germination (48 to 96 h).

### ATG8 co-localizes with LDs in the cotyledon cells of seedlings

In this study, the autophagic marker ATG8b protein was found to co-localize with LDs during the course of seed germination. Nevertheless, this co-localization was restricted to a limited pool of LDs and intensified under continuous dark conditions. After determining localization of LDs within the vacuoles by electron microscopy, we did not observe association of macroautophagic membrane structures around the LDs. A similar pattern of co-localization was also previously reported for DsRed-ATG8e-labeled puncta and LDs in Arabidopsis leaves under dark-induced starvation by Fan et al. (2019). Moreover, in the same study, no macroautophagic membrane structures were found around LDs localized inside the vacuoles, suggesting that in Arabidopsis leaves degradation of LDs occurs via the microlipophagy pathway. Interestingly, another study on Arabidopsis leaves showed that GFP-ATG8-labeled structures localized at photodamaged chloroplasts but they never completely surrounded the entire chloroplast (Nakamura et al. 2018). Based on their results, the authors proposed that ATG8-associated structures are essential for triggering chlorophagy and are also directly involved in the tonoplast-mediated sequestering of chloroplasts (Nakamura et al. 2018). It cannot be excluded that similar ATG8-dependent mechanisms govern the LD trafficking between the cytoplasm and the vacuoles prior to their degradation during seed germination.

Our immunoblotting studies revealed that during seed germination ATG8–PE conjugates are abundantly present in the protein fraction isolated from LDs, indicating that ATG8–PE is indeed associated with LDs. Accumulation of the lipidated ATG8 form has also been reported for Arabidopsis leaves under darkness-induced starvation (Fan et al. 2019). In the same study, it was also found that degradation of LDs via a microlipophagy-resembling process relies on the core ATG proteins, including ATG2 and ATG5 (Fan et al. 2019). Interestingly, the latter protein has been shown to be crucial for lipidation of ATG8 (Martens and Fracchiolla 2020) as well as for maintaining TAG degradation, as shown in *atg5-1* and *atg5-3* mutants of Arabidopsis (Avin-Wittenberg et al. 2015).

### ATG8 interacts with structural proteins of LDs

To investigate which of the structural LD proteins could directly interact with ATG8s, we searched for AIMs in Arabidopsis caleosins and OLE1. As described by Marshall et al. (2019), the latter protein was previously shown to bind the LIR/AIM-docking site on ATG8. Interestingly, 2 WxxL motifs with a very low PSSM value identified in the OLE1 sequence by the iLIR tool are most likely not functional AIMs, as AIMs with PSSM higher than 13 are considered to be reliable predictions (Kalvari et al. 2014; Klionsky et al. 2016). It should, however, be noted that numerous ATG8-interacting plant proteins that contain AIMs with a PSSM score below 13 have also been identified (Liu et al. 2021). Similar to Marshall et al. (2019), we also confirmed binding of OLE1 to ATG8b and ATG8h proteins in the split-ubiquitin 2-hybrid assays. These data suggest that interaction between OLE1 and ATG8 may occur via AIM, but its presence has yet to be determined using other algorithms, in particular those that allow identification of noncanonical AIMs (Ibrahim et al. 2023).

Interestingly, we found putative AIMs (AIM1 and AIM2) with relatively high PSSM scores (between 7 and 17) in all of the analyzed caleosins. Along this line, we also confirmed that CLO1, CLO2, and CLO3 interact with ATG8 proteins by using split-ubiquitin 2-hybrid assays. The stronger interaction between CLO1 and ATG8b or ATG8h compared with CLO2 may explain the loss of the microlipophagy phenotype that was observed only in *clo1* mutants. Notably, the interaction between CLO3 and ATG8b suggests that this leaf-specific caleosin may be important for LD degradation by microlipophagy in Arabidopsis leaves.

To gain more insights into the role of AIMs in the binding of CLO1 to ATG8, we generated several CLO1 mutants with deletions of both AIM1 and AIM2 motifs. While the deletion of the C-terminal AIM2 caused a slight decrease in the binding of CLO1 to both ATG8b and ATG8h, the absence of the transmembrane-localized AIM1 almost completely abolished the interaction between these proteins, suggesting that AIM1 may play a role in the binding of CLO1 to ATG8b and ATG8h. To date, several functional AIMs in transmembrane regions have been reported in diverse plant proteins (Stephani and Dagdas 2020; Zhang et al. 2020). Similarly to AIMs in maize (*Zea mays*) reticulon proteins Rtn1/2 (Zhang et al. 2020), AIM1 in caleosins seems to be located close to the cytoplasmic face of the LDs lipid monolayer (Chen et al. 1999) and therefore can be partly accessible to ATG8 proteins, even when caleosins are still embedded in the LD membrane. Partially impaired binding to ATG8 observed for the N-terminal truncated mutant of CLO1, despite the presence of the full-length AIM1, might be the result of structural changes of the protein affecting CLO1 targeting and its orientation at the LD membrane. Indeed, N-terminal truncated mutants of Arabidopsis CLO1 (up to 95 amino acids; Purkrtová et al. 2015) as well as the mutated sesame (*Sesamum indicum*) seed caleosin, structurally similar to Arabidopsis CLO1, which lacks the helical subdomain (residues 100 to 115; Chen and Tzen 2001), have been shown to possess impaired targeting to LDs. Noticeably, all deletion mutants of CLO1 tested in our study interacted with NubWT, indicating that they were properly synthesized and stable in yeast cells. Alternatively, the interaction of caleosins with ATG8 can also be mediated by noncanonical AIMs or other motifs, such as the UIM (Marshall et al. 2019), or via not yet discovered mechanisms.

**Figure 10.**
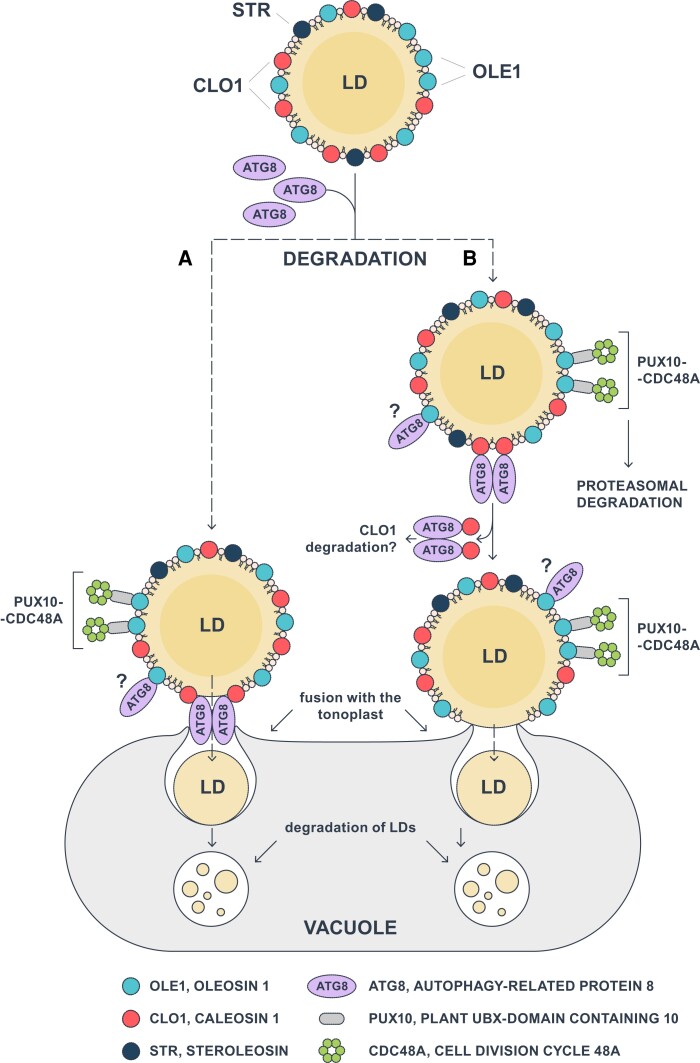
Model of CLO1-mediated microlipophagy in Arabidopsis seeds. Interaction of ATG8 with CLO1 triggers the fusion of LDs with the tonoplast via 2 possible scenarios: **A)** the complex of CLO1 and ATG8 directly mediates the fusion of the LD membrane with the tonoplast, and **B)** following its binding to CLO1, ATG8 promotes the degradation of CLO1, resulting in direct interaction and fusion between LDs and the vacuole membranes. Once transported to the vacuole, LDs undergo a gradual degradation during seed germination. The proposed pathways may coexist with other LD degradation mechanisms such as dislocation and degradation of oleosins mediated by PUX10 and CDC48A. In addition, OLE1 can interact with ATG8, but the nature of this interaction and its role in LD degradation have not yet been elucidated. ATG8, AUTOPHAGY-RELATED PROTEIN 8; CDC48A, CELL DIVISION CYCLE 48A; CLO1, CALEOSIN 1; OLE1, OLEOSIN 1; LD, lipid droplet; PUX10, PLANT UBX-DOMAIN CONTAINING 10; STR, STEROLEOSIN.

In summary, our work provides additional evidence that CLO1 facilitates trafficking of LDs to the vacuole for their degradation. We also demonstrate that Arabidopsis caleosins CLO1, CLO2, and CLO3 are ATG8 interactors. Our future goal is to explore if AIM-deficient caleosins from Arabidopsis interact with ATG8 in a physiological context. Nevertheless, our current findings demonstrate that CLO1 interacts with ATG8 in the yeast system and that both proteins are localized at the same organelle in planta, suggesting that this interaction may be relevant for LD degradation by microlipophagy during seed germination. As illustrated in Fig. 10, we propose that the docking of ATG8 to CLO1 may promote fusion of a specific subset of LDs with the tonoplast, followed by the transfer of LDs into the vacuole, where their degradation takes place (Fig. 10A). Alternatively, the interaction between ATG8 and CLO1 may trigger degradation of LD structural proteins, enabling the fusion between the membrane of selected LDs and the tonoplast (Fig. 10B). This mechanism may operate in parallel with other pathways governing degradation of LD-coating proteins, like targeted proteasomal degradation of oleosins mediated by PUX10 and CDC48A (Fig. 10). Interestingly, the latter pathway seems to be limited to a specific subpopulation of LDs in Arabidopsis germinating seeds (Deruyffelaere et al. 2018; Kretzschmar et al. 2018). We also observed that ATG8b-EYFP co-localizes only with some LDs. It is therefore possible that, similarly to PUX10, ATG8 proteins bind to a specific subset of LDs in germinating seeds, triggering their degradation by microlipophagy. Although further studies will be required to determine the molecular mechanisms behind the interaction of LDs with the vacuoles in plant cells, the crucial role of a specific caleosin CLO1 in this process is now better established in germinating seeds. Consequently, our findings open further avenues of research exploring integrated aspects of lipid mobilization in diverse plant tissues.

## Materials and methods

### Plant materials and growth conditions

The seeds of Arabidopsis (*A. thaliana*) T-DNA insertion lines for CLO1 (*Atclo1-1*, Poxleitner et al. 2006) and CLO2 (SALK_046559) in the Columbia 0 (Col-0) accession were obtained from the Nottingham Arabidopsis Stock Centre (NASC). The Col-0 ecotype was used as a wild-type control. The *clo1* mutant was previously described by Poxleitner et al. (2006). Homozygous lines were identified by PCR using the left border primer of the T-DNA insertion and the allele-specific primers listed in Supplemental Table S3. Arabidopsis lines containing the indicator for the tonoplast, γ-TIP, generated with the vac:CFP, were obtained from NASC under NASC stock number: N16256. For soil-grown plants, seeds were sown on soil, cold-stratified for 48 h, and transferred to a growth chamber (22 °C, 16 h light/8 h dark, 120 to 150 *µ*mol m^−2^ s^−1^). For plate-grown plants, seeds were surface-sterilized with 30% (v/v) bleach solution, washed 4 times with distilled water, and placed on agar-solidified half strength Murashige and Skoog (0.5×MS) medium (without sucrose; -Suc). After stratification at 4 °C in the dark for 2 d, the plates were incubated at 22 °C under a 16 h light/8 h dark photoperiod, continuous dark conditions (24 h of dark), or continuous light conditions (24 h of light).

### Concanamycin A treatment

For ConcA (Merck KgaA, Darmstadt, Germany) treatment, seeds were surface-sterilized as described above and placed on 0.5×MS medium (without sucrose; -Suc) or 0.5×MS medium -Suc supplemented with 1 *μ*m ConcA or DMSO (1% v/v) (control solvent). After stratification at 4 °C in the dark for 2 d, plates were kept at 22 °C under a 16 h light/8 h dark photoperiod. Images of the plates were taken after 3 and 9 d of growth. On the 9th day, the samples were collected for confocal imaging and lipid analysis.

### Seed phenotypic analysis

Mature seeds were collected and then photographed using the Olympus SZX12 stereomicroscope with R6 Retiga digital camera (Qimaging) Length and width measurements were performed using ImageJ software (Schneider et al. 2012).

### Total RNA extraction and cDNA synthesis

Total RNA was extracted from 100 mg of Arabidopsis siliques, germinating seeds or rosette leaves using Spectrum Plant Total RNA Kit (Merck KgaA), according to the manufacturer’s instructions. Total RNA (1 *µ*g) was used for cDNA synthesis using RevertAid H Minus First Strand cDNA Synthesis Kit and Oligo(dT)18 primer (Thermo Fisher Scientific, Bremen, Germany).

### Construction of plasmids and Arabidopsis transformation

The coding sequence for *CLO1* and *ATG8b* were amplified from cDNA using Phusion High-Fidelity DNA polymerase (Thermo Fischer Scientific) and attB primers listed in Supplemental Table S4. The amplified sequences were inserted into the pDONR207 vector using Gateway BP Clonase II Enzyme mix (Thermo Fischer Scientific) and then transferred into the pEarleyGate 104 (EYFP at the N-terminus) vector (Earley et al. 2006) using Gateway LR Clonase II Enzyme mix (Thermo Fischer Scientific). The resulting plasmids were introduced to *Agrobacterium tumefaciens* strain GV3101 and used for transformation of Arabidopsis via floral dipping (Clough and Bent 1998). T1 transgenic lines were selected by spraying 7-d-old seedlings with 0.05% (w/v) Basta solution and surviving plants were subjected to Western blot analysis for the detection of EYFP (see below). T3 transgenic lines were used for microscopic analyses (see below).

### Total protein isolation

Plant material was homogenized with a TissueLyser II (Qiagen) and protein extraction buffer [100 mm Tris pH 8.0, 2 mm phenylmethylsulfonyl fluoride, 2% (v/v) β-mercaptoethanol, 4% (w/v) SDS], was added (0.5 mL) to the samples. The samples were then heated for 3 min at 80 °C and centrifuged at 13,000*g* for 5 min. The supernatant was transferred to a new tube and used for SDS–PAGE.

### Isolation of LDs and LD-associated protein

LDs were isolated as described in Rudolph et al. (2011) with modifications. Briefly, mature and/or germinating seeds (24 and 48 h) of Arabidopsis (Col-0, *clo1*, *clo2*, and *clo1clo2*) were homogenized at 4 °C in grinding buffer [15% (w/v) sucrose, 150 mm Tris-HCl, pH 7.5, 10 mm KCl, 1.5 mm EDTA, 0.1 mm MgCl_2_, and 5 mm dithiothreitol]. The homogenates were centrifuged at 100,000*g* for 60 min at 4 °C. The floating fat pad was collected and resuspended in cold flotation buffer [10% (w/v) sucrose, 50 mm Tris-HCl buffer, pH 7.5, 10 mm KCl, 1.5 mm EDTA, and 0.1 mm MgCl_2_]. After centrifugation at 100,000*g* for 60 min at 4 °C, the resulting fat pad was collected and homogenized in 0.2 m borate buffer, pH 8.25. Proteins were precipitated at −20 °C, overnight using acetone/ethanol (4:1, v/v), followed by washing with 80% ethanol.

### SDS–PAGE and immunoblotting

Protein samples (25 *µ*g) were mixed with 2× Laemmli sample buffer and boiled for 5 min. Then, proteins were subjected to 12% SDS–PAGE and transferred onto a nitrocellulose membrane. Membranes were incubated for 1 h in blocking solution containing 1% (w/v) nonfat dry milk in Tris-buffered saline plus Tween 20 [TBST, 20 mm Tris-HCl pH 7.5, 150 mm NaCl and 0.05% (v/v) Tween 20, pH 7.5]. Membranes were subjected to immunoblot analysis using primary antibodies against CLO1 [diluted 1:1,000 (Næsted et al. 2000)], oleosin [diluted 1:1,000 (Rudolph et al. 2011)], ATG8 (diluted 1:500, catalog no. AS 142769; Agrisera), GFP (diluted 1:1,000, catalog no. 11814460001; Roche) or α-tubulin (diluted 1:5,000, catalog no. AS10680; Agrisera). Alkaline phosphatase-conjugated anti-rabbit or anti-chicken IgG (diluted 1:30,000; Merck) or horseradish peroxidase-conjugated anti-rabbit or anti-mouse IgG secondary antibody (diluted 1:50,000; Merck) served as a secondary antibody. For the alkaline phosphatase system, antigens were visualized on nitrocellulose membrane as purple-brown bands in the presence of nitrotetrazolium blue chloride and 5-bromo-4-chloro-3ʹ-indolyphosphate p-toluidine. The signal from horseradish peroxidase-conjugated antibodies was detected by using western ECL Substrate (Amersham), and the signal was visualized using a ChemiDoc MP Imaging System (Bio-Rad). Densitometric analysis was performed by using Image Lab 5.0 software (Bio-Rad).

### Co-IP assay

LD-associated proteins were co-immunoprecipitated using the Dynabeads co-immunoprecipitation kit (Thermo Fisher Scientific) and an anti-CLO1 antibody according to the manufacturer’s protocol for Western blot. The obtained Dynabeads Co-IP complexes were processed, according to the manufacturer’s protocol. Proteins bound to Dynabeads were eluted with 40 *µ*L of elution buffer and analyzed by SDS–PAGE. For immunoblotting, the membrane was cut between the 25 and 15 kDa marker bands, and the upper part of the membrane was probed with the anti-CLO1 antibody while the lower part was probed with the anti-ATG8 antibody as described above. The experiment was repeated in triplicate.

### Yeast split-ubiquitin assay

The direct protein–protein interactions were analyzed using the mating-based split-ubiquitin system (Obrdlik et al. 2004; Grefen et al. 2007). The coding regions of the selected ATG8, caleosin, and oleosin genes were amplified using Phusion High-Fidelity DNA polymerase (Thermo Fischer Scientific) and primers listed in Supplemental Table S5. Site-directed mutagenesis of AIMs was performed using a Q5 site-directed mutagenesis kit (New England BioLabs, Frankfurt am Main, Germany) using primers listed in Supplemental Table S5, according to the manufacturer’s protocol. AIMs were identified in caleosin sequences and OLE1 using the iLIR tool [https://ilir.warwick.ac.uk, Kalvari et al. (2014)]. PCR products were inserted into pMetYCgate or pNXgate32-3HA vectors by in vivo recombination in THY.AP4 and THY.AP5 yeast (*Saccharomyces cerevisiae*) strains, respectively, as described in Grefen et al. (2007). Transformants were selected on synthetic complete (SC) media lacking leucine (THY.AP4) or tryptophan (THY.AP5). The bait or prey vectors were isolated from several yeast clone cultures and transformed into *Escherichia coli* for re-isolation and sequencing. After sequence confirmation, the bait or prey vectors were again transformed into THY.AP4 and THY.AP5 strains, respectively. The obtained colonies were subsequently used for mating in appropriate combinations. Diploids were selected by replica plating on SC medium lacking leucine, tryptophan, uracil, and methionine. In order to detect the protein–protein interactions, diploid colonies were grown on liquid SC medium lacking leucine, tryptophan, uracil, and methionine overnight at 28 °C. To confirm the interactions, yeast cells (100 *μ*L) were harvested and diluted in sterile water to an OD_600_ of 1.0, 0.1, and 0.01, and 4 *μ*L of each dilution was spotted onto a selective medium and grown for 1 to 3 d at 28 °C. The selected interactions were verified by quantitative β-galactosidase assays using Beta-Glo Assay System (Promega, Madison, WI, USA). Briefly, 10 *μ*L of the overnight culture diluted to an OD_600_ of 0.02 was mixed with 10 *μ*L of Beta-Glo Reagent and the assay was performed according to the manufacturer’s protocols. X-Gal overlay assay was used as an additional method to confirm protein–protein interactions and was carried out as described in Grefen et al. (2007). The soluble wild-type NubI (the pNubWT-Xgate vector) and NubG (the empty pNXgate32-3HA vector) were used as a positive and a negative control, respectively. Additionally, pMetYCgate carrying *AtOLE1* was used as a positive control (Marshall et al. 2019).

### Fatty acid analysis

Lipids were extracted from mature seeds and seedlings germinated on MS medium as described by Marmon et al. (2017). Briefly, 1 mL of methanolic solution containing 2.75% (v/v) H_2_SO_4_ (95% to 97%), 2% (v/v) dimethoxypropan, and 2% (v/v) toluene was added to the plant material. Samples were shaken at 80 °C for 45 min and washed with 1 mL *n*-hexane and 1.2 mL of saturated NaCl solution. After centrifugation at 1,000*g* for 10 min, the upper phase was collected and the samples were dried under nitrogen streaming. Fatty acid methyl ester analysis was performed according to Marmon et al. (2017). For quantification, an internal standard of tripentadecanoate (tri-15:0) was added to each sample before methylation. Raw data from lipid analysis are provided in Supplemental Data Set 1.

### Confocal laser scanning microscopy

LDs were observed using BODIPY 505/515. Plant material was incubated with 1 mm BODIPY 505/515 for 30 min and washed with phosphate-buffered saline (pH 7.2). Samples were observed under a Leica TCS SP5 or Olympus F3000 confocal microscope with excitation at 505 nm and emission at 515 nm for BODIPY (diode laser 488 nm, intensity 0.1%, excitation bandwidth 505/5, emission bandwidth 515/5, gain 1.0). EYFP fusion proteins were observed with excitation at 513 nm and emission at 527 nm (diode laser 488 nm, intensity 1.25%, excitation bandwidth 494/40, emission bandwidth 540/30, gain 1.0). CFP fusion protein was observed with excitation at 405 nm and emission at 485 nm (diode laser 405 nm, intensity 3%, excitation bandwidth 435/40 emission bandwidth 485/50, gain 1.0). Chlorophyll autofluorescence was captured using excitation at 633 and emission at 700 nm (diode laser 640 nm, intensity 0.5%, excitation bandwidth 640, emission bandwidth 700/30, gain 1.0). A total of 30 randomly chosen samples were used for the observation, and all images were processed using ImageJ software (Schneider et al. 2012).

### Transmission electron microscopy

Cotyledons were collected after 48 or 72 h of seed in vitro germination. Sample preparation was carried out as described in Zienkiewicz et al. (2020b). Briefly, the samples were fixed in 2.5% (v/v) glutaraldehyde in 0.1 m cacodylate buffer (pH 7.2) overnight at 4 °C. Next, the samples were postfixed in OsO_4_ at room temperature for 1 h, dehydrated in ethanol series, and embedded in Araldite-Epon resin (Araldite 502/Embed 812 Kit; Electron Microscopy Sciences, Science Services GmbH, München, Germany). Ultrathin sections were observed with a JEOL 1011 TEM (Japan Electron Optics Laboratories Germany GmbH, Freising, Germany) at 80 kV. Images were processed using ImageJ software (Schneider et al. 2012).

### Sequence analysis

The protein sequences of Arabidopsis caleosins with the following accession numbers, CLO1 (O81270), CLO2 (Q9FLN9), CLO3 (O22788), CLO4 (Q9CAB7), CLO5 (B3H7A9), CLO6 (Q9CAB8), CLO7 (F4I4P8), and CLO8 (A0A178V895), were retrieved from the UniProt database (www.uniprot.org) and aligned by ClustalW using MEGA-X software (Kumar et al. 2018). In this paper, we followed the nomenclature of Arabidopsis caleosins proposed by Shen et al. 2014 (Supplemental Table S6).

### Statistical analyses

Statistical analyses were performed using R 4.2 software and Statistica 13.1 software (StatSoft, Poland). Statistically significant differences were determined by 1-way ANOVA, followed by post hoc Tukey’s test, or by a 2-tailed Student’s *t*-test (Supplemental Data Set 2).

### Accession numbers

Sequence data from this article can be found in the GenBank/EMBL data libraries under accession numbers, CLO1 (O81270), CLO2 (Q9FLN9), CLO3 (O22788), CLO4 (Q9CAB7), CLO5 (B3H7A9), CLO6 (Q9CAB8), CLO7 (F4I4P8), CLO8 (A0A178V895), and OLE1(P29525).

## Supplementary Material

kiad471_Supplementary_Data

## Supplemental data

The following materials are available in the online version of this article.

**Supplemental Figure S1.** Characterization of T-DNA insertion *clo1* and *clo2* mutant lines of Arabidopsis.

**Supplemental Figure S2.** The effect of loss of function of CLO1 and CLO2 on Arabidopsis seed morphometric parameters.

**Supplemental Figure S3.** Fatty acid analysis in Col-0 and caleosin mutants during seed germination under long day conditions.

**Supplemental Figure S4.** Fatty acid analysis of Col-0 and caleosin mutants during seed germination under continuous dark conditions.

**Supplemental Figure S5.** Expression of *CLO1-EYFP* in Arabidopsis transgenic plants.

**Supplemental Figure S6.** Expression of *ATG8b-EYFP* in Arabidopsis transgenic plants.

**Supplemental Figure S7.** CLO1 and ATG8 abundance in wild-type plants and caleosin mutants.

**Supplemental Figure S8.** Multiple alignment of Arabidopsis caleosins.

**Supplemental Figure S9.** Interaction between caleosins and ATG8 proteins.

**Supplemental Figure S10.** Representative co-immunoprecipitation assay showing the binding of CLO1 to ATG8.

**Supplemental Table S1.** Putative AIMs in caleosins from Arabidopsis.

**Supplemental Table S2.** Putative AIMs with a low PSSM score in CLO1 and OLE1.

**Supplemental Table S3.** Gene-specific primers (LP, left primer; RP, right primer) and T-DNA left border primers (LBb1.3 and DSPM1) used for the genotyping of plants.

**Supplemental Table S4.** Gene-specific primers (F, forward primer; R, reverse primer) used for the construction of the *35S::EYFP* (pEarleyGate 104) plasmids.

**Supplemental Table S5.** Gene-specific primers (F, forward primer; R, reverse primer) used for the construction of the yeast plasmids.

**Supplemental Table S6.** The nomenclature of caleosins from Arabidopsis used in the literature.

**Supplemental Data Set S1.** Raw data from lipid analysis.

**Supplemental Data Set S2.** ANOVA and Student’s *t*-test results.

## Dive Curated Terms

The following phenotypic, genotypic, and functional terms are of significance to the work described in this paper:

CDC48 Gramene: AT3G09840
CDC48 Araport: AT3G09840

RAV1 Gramene: AT1G13260
RAV1 Araport: AT1G13260

SDP1 Gramene: At5G04040
SDP1 Araport: At5G04040

ATG2 Gramene: AT3G51800
ATG2 Araport: AT3G51800

ATG8b Gramene: AT4G04620
ATG8b Araport: AT4G04620

PUX10 Gramene: AT4G10790
PUX10 Araport: AT4G10790

CLO1 Gramene: At4G26740
CLO1 Araport: At4G26740

CLO2 Gramene: AT5G55240
CLO2 Araport: AT5G55240
