# Supplementary material for: CALEOSIN 1 interaction with AUTOPHAGY-RELATED PROTEIN 8 facilitates lipid droplet microautophagy in seedlings
Source: Plant Physiol. 2023 Aug 24;193(4):2361–80. doi: 10.1093/plphys/kiad471 (PMC10663143; doi:10.1093/plphys/kiad471)
Supplement: kiad471_Supplementary_Data [file kiad471_supplementary_data.zip › PP2023-RA-00609DR1_Supplemental_Data_revised_KIAD471.pdf]

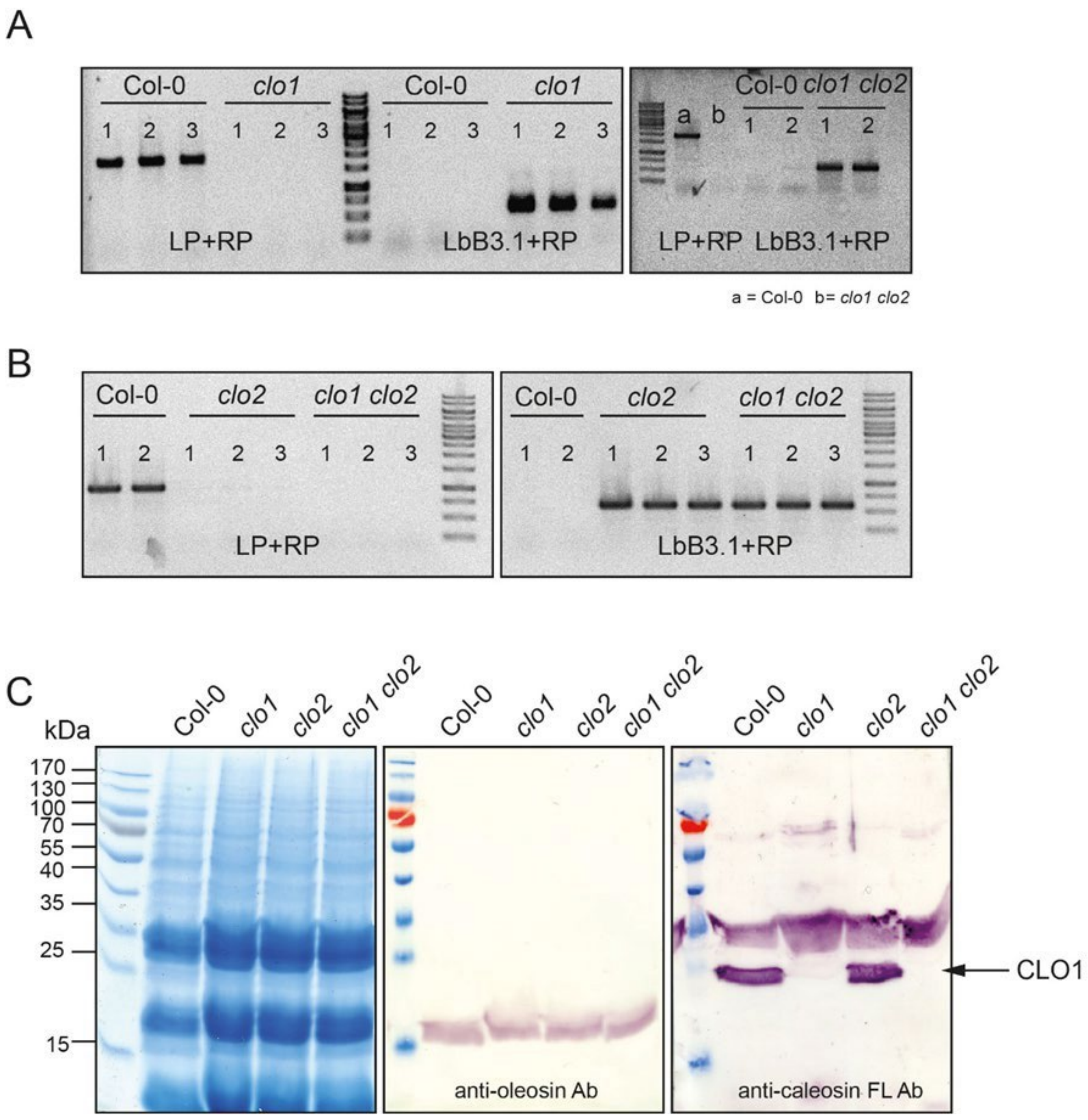

Supplemental Figure S1. Characterization of T-DNA insertion *clo1* and *clo2* mutant lines of Arabidopsis. (A) Genotyping of *clo1* and *clo1 clo2* mutants by RT-PCR using left border primer of the T-DNA insertion (LbB3.1) and *CLO1* allele-specific primers (LP and RP). (B) Genotyping of *clo2* and *clo1 clo2* mutants by RT-PCR using left border primer of the T-DNA insertion (LbB3.1) and *CLO2* allele-specific primers (LP and RP). (C) Detection of OLEOSIN 1 and CALEOSIN 1 in the total protein extract isolated from Col-0, *clo1*, *clo2* and *clo1 clo2* mature seeds.

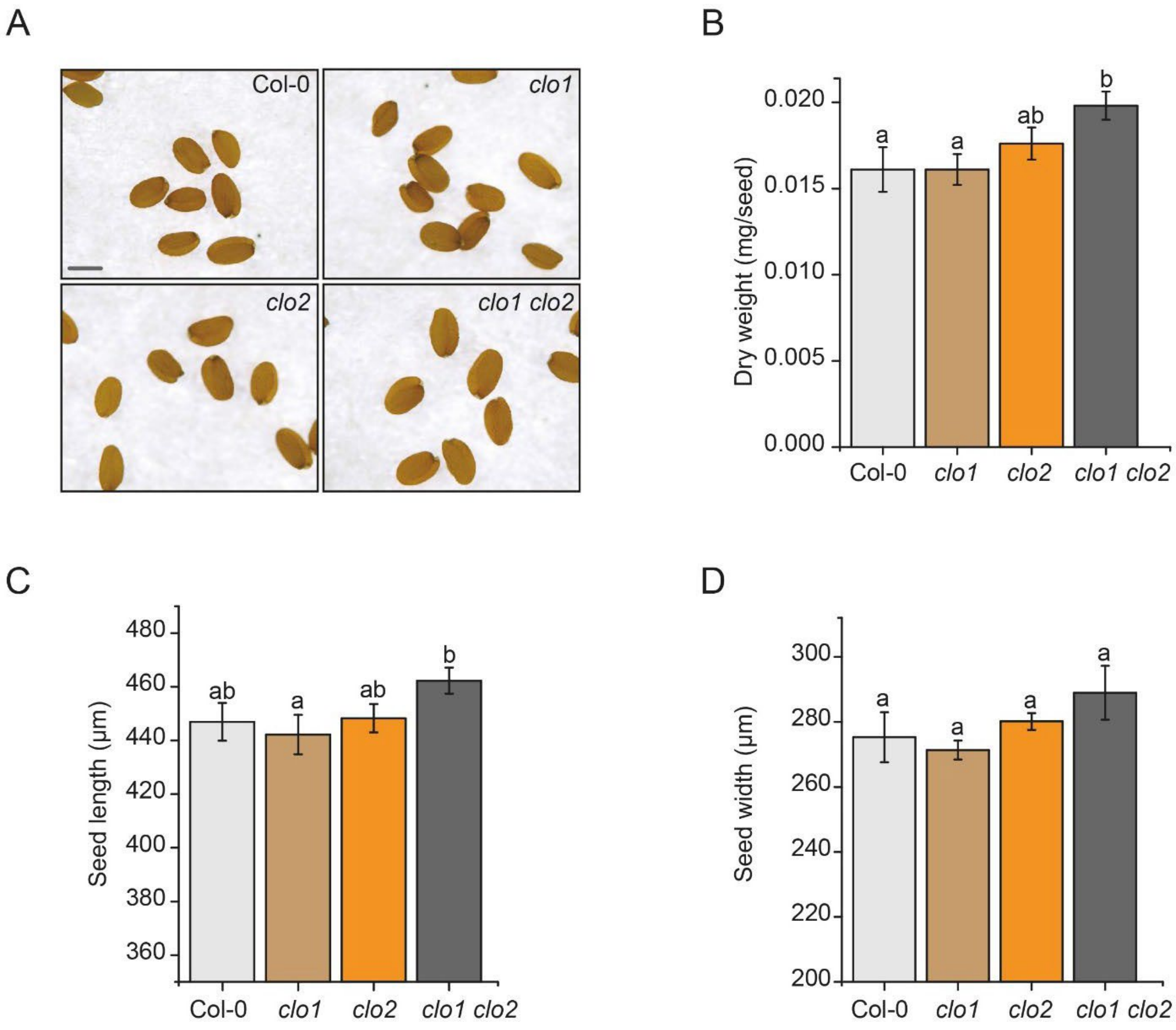

Supplemental Figure S2. The effect of loss of function of CLO1 and CLO2 on Arabidopsis seed morphometric parameters. (A) Morphology of the Col-0 and *clo1*, *clo2* and *clo1 clo2* dry mature seeds, scale bar = 500 µm. (B) Seed dry weight per one mature seed of the Col-0 and *clo1*, *clo2* and *clo1 clo2.* Data are means ± SD from 9 biological replicates (n = 300). (C) Seed length of the Col-0 and *clo1*, *clo2* and *clo1 clo2*. Data are means ± SD from 9 biological replicates (n = 150). (D) Width of mature seeds of Col-0, *clo1*, *clo2* and *clo1 clo2*. Data are means ± SD from 9 biological replicates (n = 150). Statistical analysis was performed by one-way ANOVA with Tukey's post hoc test. Different letters indicate significant differences with $P < 0.05$.

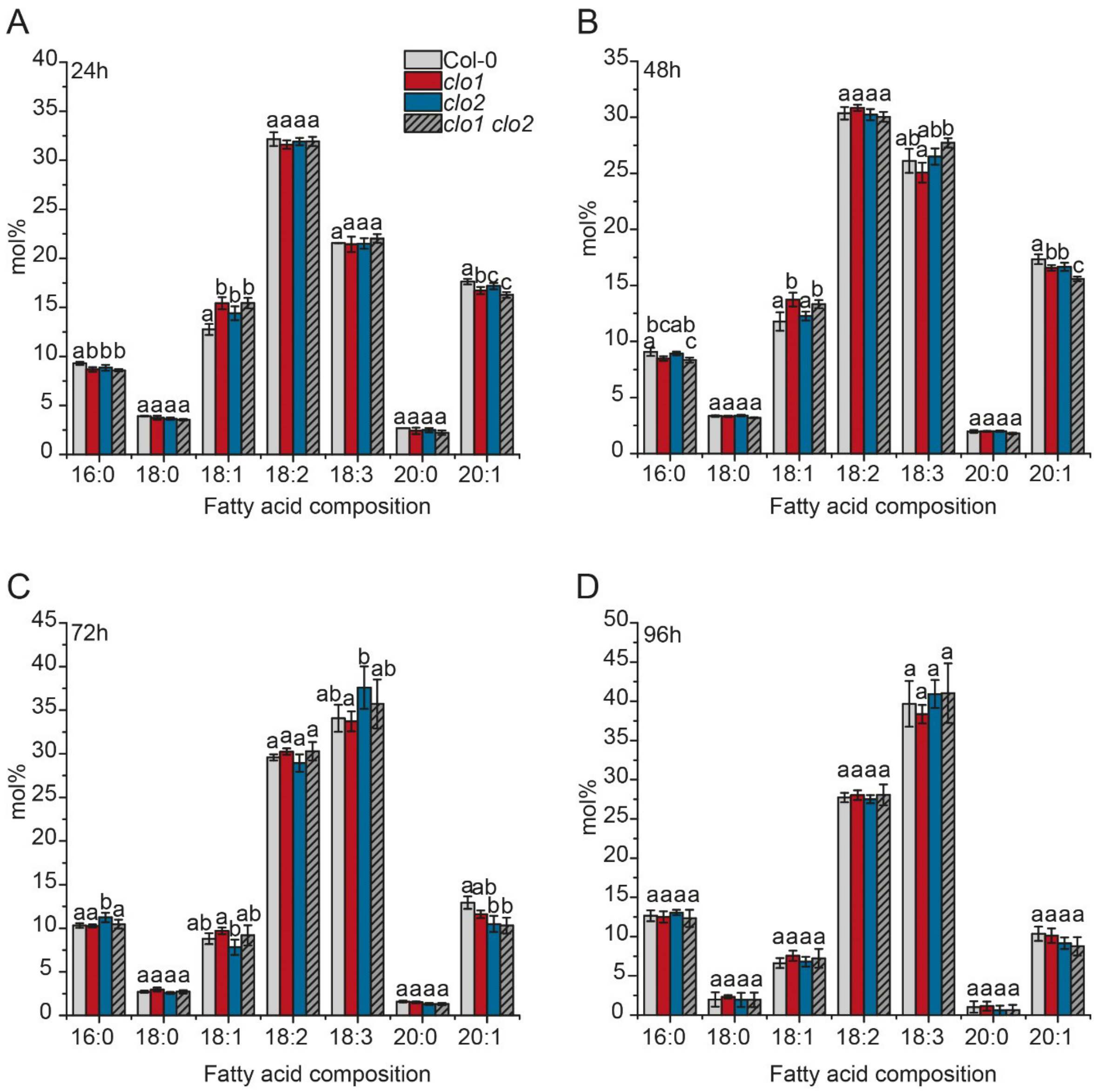

Supplemental Figure S3. Fatty acid analysis in Col-0 and caleosin mutants during seed germination under long day conditions. (A–D) Changes in FA (mol%) composition between Col-0, *clo1*, *clo2*, *clo1 clo2* after 24 h (A), 48 h (B), 72 h (C), and 96 h (D) of seed germination. Data are means ± SD from two independent experiments of six biological replicates (n = 6). Statistical analysis was performed by one-way ANOVA with Tukey‘s post hoc test. Different letters indicate significant differences with P < 0.05. 16:0, palmitic acid; 18:0, stearic acid; 18:1, oleic acid; 18:2, linoleic acid; 18:3, linolenic acid; 20:0, arachidic acid; 20:1, eicosenoic acid.

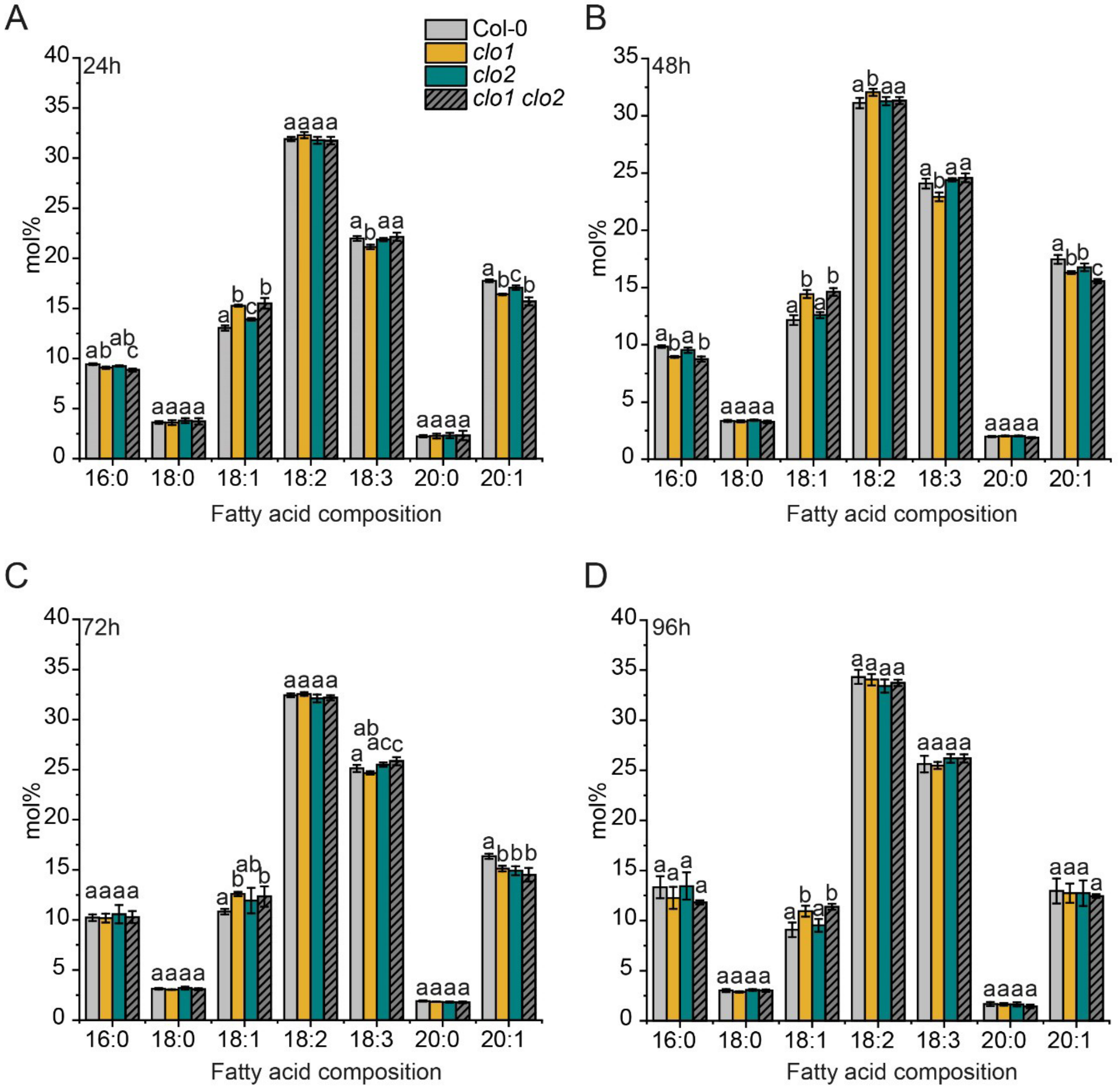

Supplemental Figure S4. Fatty acid analysis of Col-0 and caleosin mutants during seed germination under continuous dark conditions. (A–D) Changes in FA (mol%) composition between Col-0, *clo1*, *clo2*, *clo1 clo2* after 24 h (A) 48 h (B), 72 h (C) and 96 h (D) of seed germination. Data are means ± SD from two independent experiments of six biological replicates (n = 6). Statistical analysis was performed by one-way ANOVA with Tukey's post hoc test. Different letters indicate significant differences with $P < 0.05$. 16:0, palmitic acid; 18:0, stearic acid; 18:1, oleic acid; 18:2, linoleic acid; 18:3, linolenic acid; 20:0, arachidic acid; 20:1, eicosenoic acid.

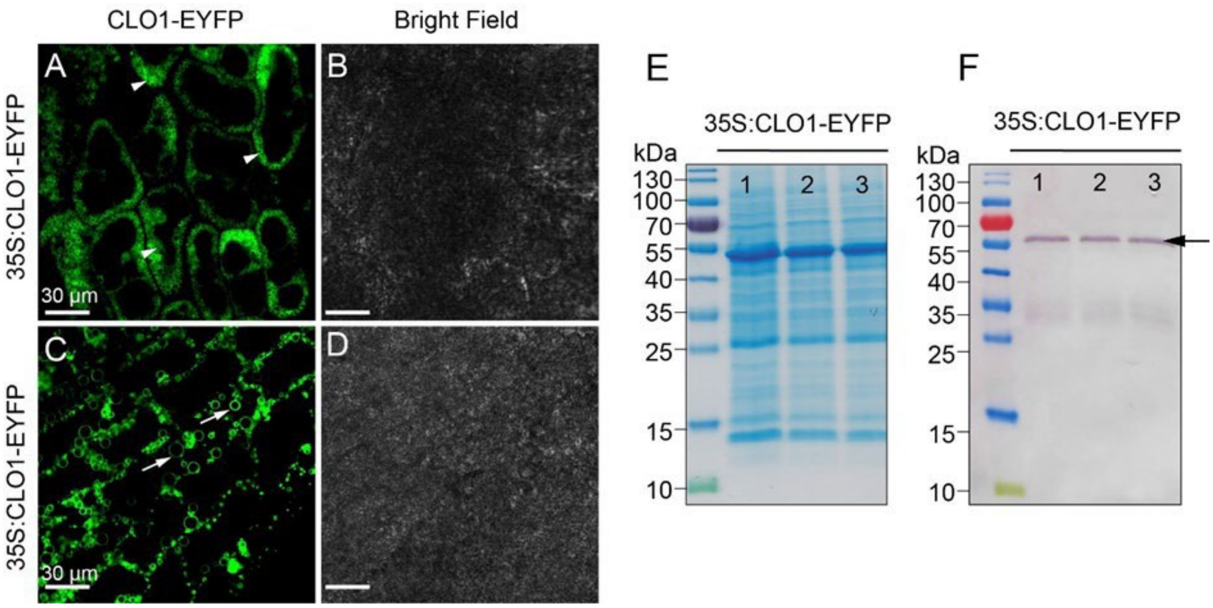

Supplemental Figure S5. Expression of *CLO1-EYFP* in Arabidopsis transgenic plants. (A–D) Representative CLSM images of Arabidopsis seeds expressing the *CLO1-EYFP* (green) construct under the 35S promoter after 24 h (A–B) and 48 h (C–D) of seed germination. Arrowheads indicate CLO1-EYFP localized to cytoplasmic LDs. Arrows indicate the vacuolar pool of LDs. (E) Coomassie brilliant blue stained SDS-PAGE gel of total proteins from the 4-week-old rosette leaves of three Arabidopsis plants (lines 1, 2, 3) expressing *CLO1-EYFP*. (F) Detection of CLO1-EYFP by immunoblotting in 4-week-old rosette leaves of three Arabidopsis transgenic plants (lines 1, 2, 3). The arrow indicates CLO1-EYFP. CLO1, CALEOSIN 1; EYFP, enhanced yellow fluorescent protein.

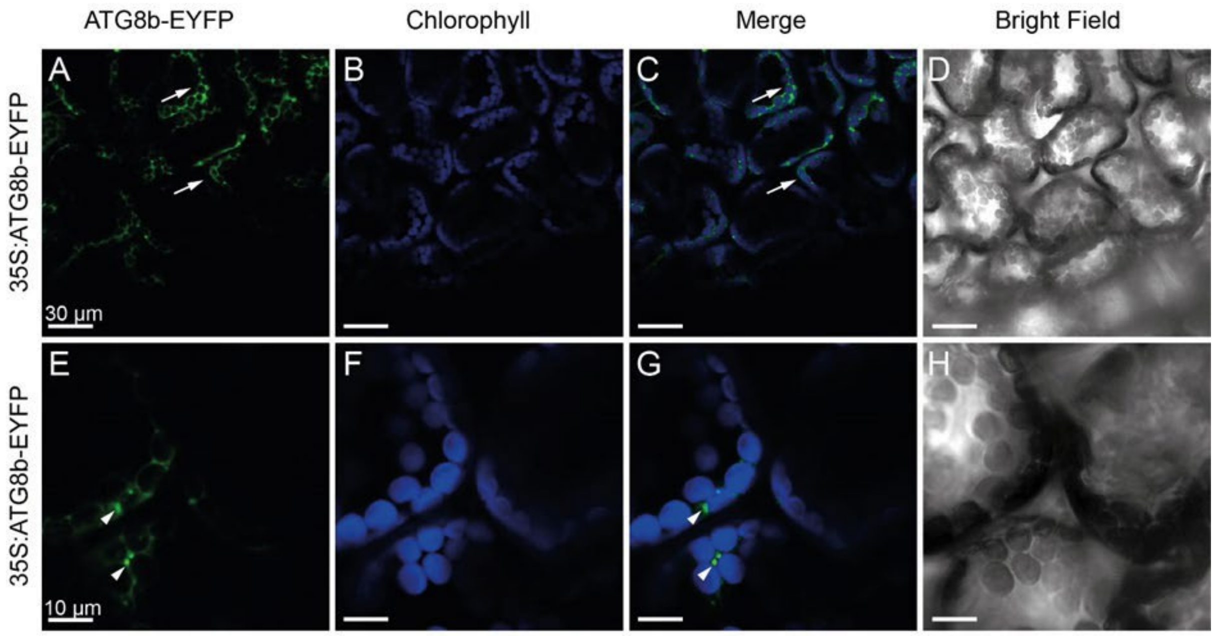

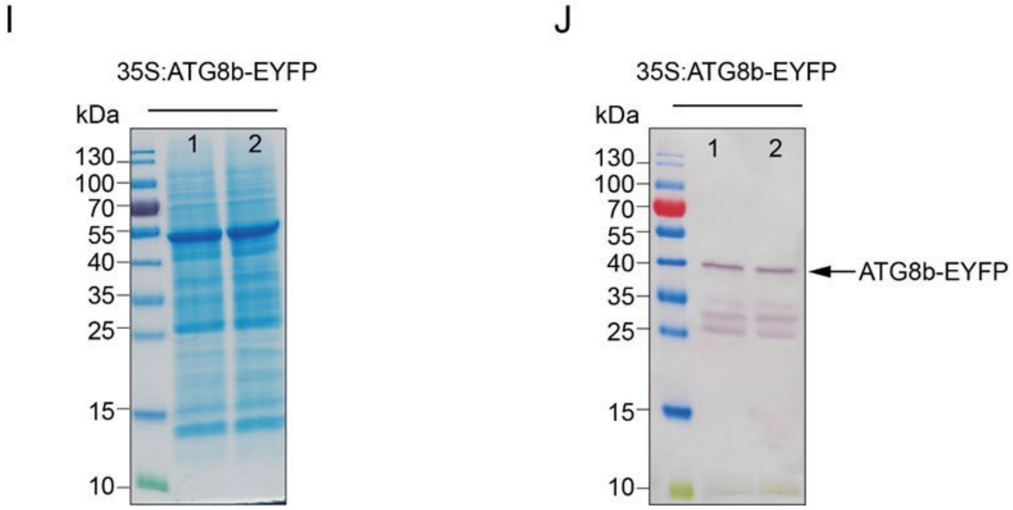

Supplemental Figure S6. Expression of *ATG8b-EYFP* in Arabidopsis transgenic plants. (A-H) Representative CLSM images of 4-week-old rosette leaves of Arabidopsis expressing the *ATG8b-EYFP* construct (green) under the 35S promoter. Arrows indicate a cytoplasmic pool of ATG8b and arrowheads indicate ATG8b-labeled autophagic structures. (I) SDS-PAGE gel of total proteins isolated from 4-week-old rosette leaves and stained by Coomassie blue. (J) Immunoblotting detection of ATG8b-EYFP by using an anti-GFP antibody. Lines 1 and 2 correspond to two biological replicates. ATG8b, AUTOPHAGY-RELATED PROTEIN 8b; EYFP, enhanced yellow fluorescent protein.

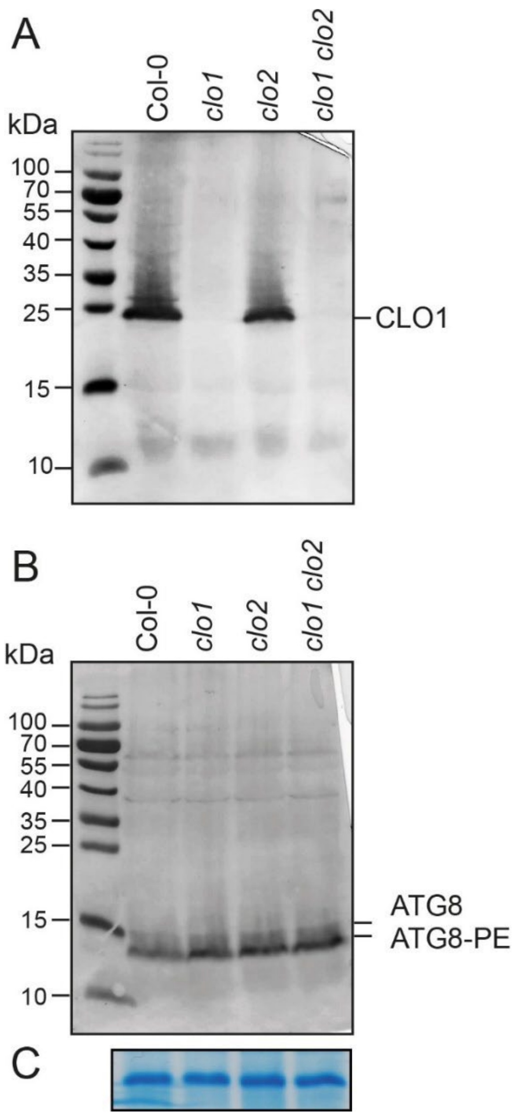

Supplemental Figure S7. CLO1 and ATG8 abundance in wild-type plants and caleosin mutants. Representative immunoblotting for CLO1 (A) and ATG8 (B) detected in LD protein fraction extracted at 24 h of seed *in vitro* germination. (C) SDS-PAGE loading control stained with Coomassie blue. CLO1, CALEOSIN 1; ATG8, AUTOPHAGY-RELATED PROTEIN 8; PE, phosphatidylethanolamine.

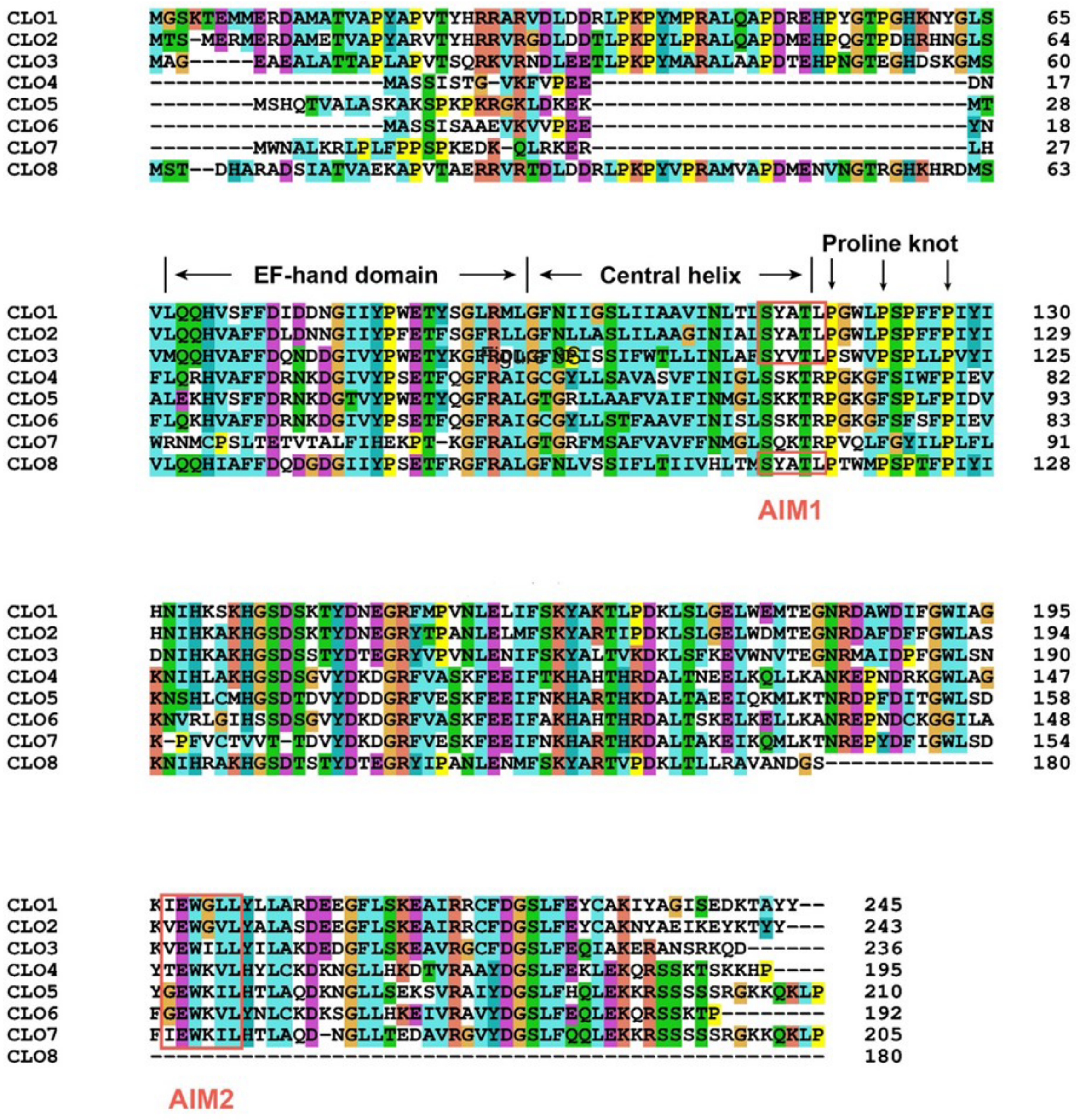

Supplemental Figure S8. Multiple alignment of Arabidopsis caleosins. The protein sequences of Arabidopsis caleosins with the following accession numbers: CLO1 (O81270); CLO2 (Q9FLN9); CLO3 (O22788); CLO4 (Q9CAB7); CLO5 (B3H7A9); CLO6 (Q9CAB8); CLO7 (F4I4P8); CLO8 (A0A178V895) were obtained from UniProt database (www.uniprot.org) and aligned by ClustalW using MEGA-X software. The EF-hand motif, the central helix, and the proline knot are shown above the sequences according to Purkrtova et al. (2007). Putative ATG8-Interacting Motifs (AIMs), AIM1 and AIM2, are highlighted by pink frames.

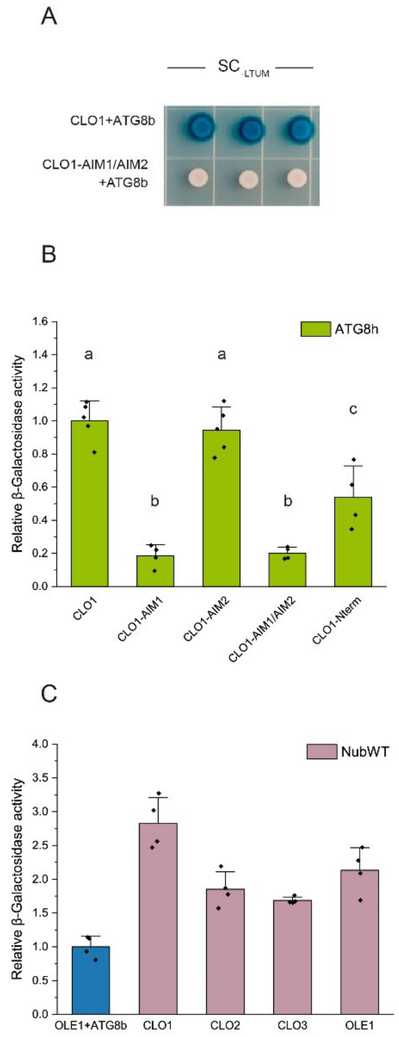

Supplemental Figure S9. Interaction between caleosins and ATG8 proteins. (A) X-Gal overlay assay for detection of β-galactosidase activity. Diploid yeasts were spotted on the SC medium without Leu, Trp, Ura, and Met (SC-LTUM). (B) Quantitative β-galactosidase activity assay of Cub fusions of CLO1 variants shown in Figure 9 (bait) with the NubG fusion of ATG8h (prey). Data are means ± SD from 4-5 independent yeast transformants. The β-galactosidase activity was normalized relative to the activity measured for the interaction between CLO1 and ATG8h. Statistical analysis was performed by one-way ANOVA with Tukey‘s post hoc test. Different letters indicate significant ($P < 0.01$) differences between β-galactosidase activity for the tested variants. (C) Quantitative β-galactosidase activity assay for the Cub fusions of CLO1, CLO2, CLO3 or OLE1 with NubWT (positive control). Data are means ± SD from 4 independent yeast transformants. The β-galactosidase activity was normalized relative to the activity measured for the interaction between OLE1 and ATG8b (blue bar).

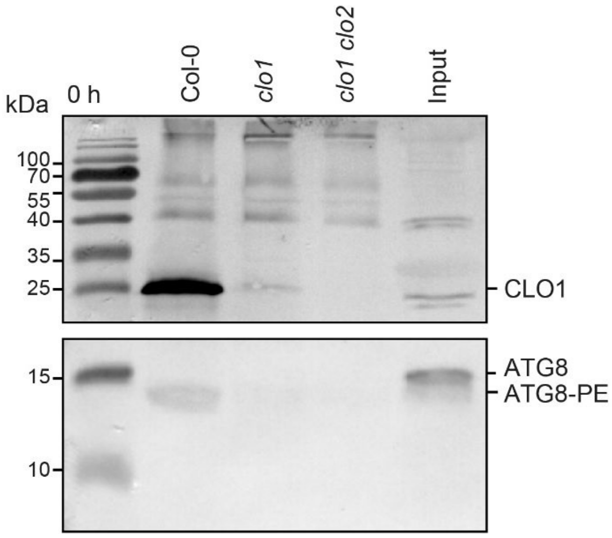

Supplemental Figure S10. Representative co-immunoprecipitation assay showing the binding of CLO1 to ATG8. Proteins extracted from LD-fraction isolated from Col-0, *clo1*, and *clo1 clo2* imbibed seeds (0 h) were immunoprecipitated using an anti-CLO1 antibody. The co-immunoprecipitated proteins were detected using an anti-CLO1 antibody (the upper part of the blot) and an anti-ATG8 antibody (the lower part of the blot).

Supplemental Table S1. Putative ATG8-Interacting Motifs (AIMs) in caleosins from Arabidopsis. Summary of AIMs with a PSSM value above 13 (or corresponding AIMs with a lower PSSM score in other caleosin sequences) identified using the iLIR tool (https://ilir.warwick.ac.uk/, Kalvari et al. (2014)); PSSM: position-specific scoring matrix; WxxL: canonical LC3-interacting region (LIR) motif; xLIR: extended LIR-motif.

Gene	Motif	LIR Sequence	Start	End	PSSM score
CLO1	WxxL	LSYATL	112	117	8
	WxxL	IEWGLL	197	202	12
CLO2	WxxL	LSYATL	111	116	8
	WxxL	VEWGVL	196	201	13
CLO3	xLIR	FSYVTL	107	112	14
	WxxL	VEWILL	192	197	16
CLO4	WxxL	TEWKVL	149	154	16
CLO5	WxxL	GEWKIL	160	165	17
CLO6	WxxL	GEWKVL	150	155	17
CLO7	WxxL	IEWKIL	156	161	15
CLO8	WxxL	MSYATL	110	115	7

Supplemental Table S2. Putative ATG8-Interacting Motifs (AIMs) with a low PSSM score in CLO1 and OLE1. Summary of AIMs with a PSSM value below 13 identified using the iLIR tool (https://ilir.warwick.ac.uk/, Kalvari et al. (2014)); PSSM: position-specific scoring matrix; WxxL: canonical LIR motif.

Gene	Motif	LIR Sequence	Start	End	PSSM score
CLO1	WxxL	APYAPV	17	22	4
	WxxL	VSFFDI	71	76	5
	WxxL	ETYSGL	86	91	8
	WxxL	LGFNII	94	99	7
	WxxL	SPFFPI	123	128	5
	WxxL	GRFMPV	149	154	4
	WxxL	DIFGWI	188	193	2
	WxxL	KIYAGI	232	237	2
OLE1	WxxL	VIFSPI	79	84	2
	WxxL	TVFSWI	111	116	0

Supplemental Table S3. **Gene specific primers (LP, left primer; RP, right primer) and T-DNA left border primers (LBb1.3 and DSPM1) used for genotyping of plants.**

Primer Name	Primer Sequence (5`3`)	Mutant Line
CLO1 LP<br>*CLO1* RP	ATGGGGTCAAAGACGGAGATGATGGAGAG<br>GGATTCTCAGACTGCTTCAATGTTCATGG	CS40119 / *Atclo1-1*<br>(Poxleitner et al., 2006)
CLO2 LP<br>*CLO2* RP	GTTCAATTCAATTGTTTCCCG<br>TTCGATTTGGACAATAATGGC	Salk_046559
LBb1.3	ATTTTGCCGATTTCGGAAC	For Salk _046559 Line
DSPM1	CTTATTTCAGTAAGAGTGTGGGGTTTTGG	For *Atclo1-1* Line<br>(Poxleitner et al., 2006)

Supplemental Table S4. Gene specific primers (F, forward primer; R, reverse primer) used for construction of the *35S::EYFP* (pEarleyGate 104) plasmids.

Primer Name	Gene ID	Primer Sequence (5`3`)
*CLO1*F attB1	At4g26740	GGGGACAAGTTTGTACAAAAAAGCAGGCTTA*ATGGGGTCA AAGACGGAGATG*
*CLO1*R attB2		GGGGACCACTTTGTACAAGAAAGCTGGGT *CGTAGTATGCTGTCTTGTCTTCACTG*
*ATG8b*F attB1	At4g04620	GGGGACAAGTTTGTACAAAAAAGCAGGCTTA*ATGGAGAA GAACTCCTTCAAG*
*ATG8b*R attB2		GGGGACCACTTTGTACAAGAAAGCTGGGT *TTAGCAGTAGAAAGATCCAC*

Supplemental Table S5. Gene-specific primers (F, forward primer; R, reverse primer) used for construction of the yeast plasmids.

Primer Name	Gene ID	Primer Sequence (5`3`)
*CLO1*F	At4g26740	ACAAGTTTGTACAAAAAAGCAGGCTCTCCAACCACC*ATGGGGTCAAAGACGGAG*
*CLO1*R		TCCGCCACCACCAACCACTTTGTACAAGAAAGCTGGGTA*GTAGTATGCTGTCTTGTCTTC*
*CLO2*F	At5g55240	ACAAGTTTGTACAAAAAAGCAGGCTCTCCAACCACC*ATGACGTCGATGGAGAGGATG*
*CLO2*R		TCCGCCACCACCAACCACTTTGTACAAGAAAGCTGGGTA*GTAGTACGTCTTGTACTC*
*CLO3*F	At2g33380	ACAAGTTTGTACAAAAAAGCAGGCTCTCCAACCACC*ATGGCAGGAGAGGCAGAGG*
*CLO3*R		TCCGCCACCACCAACCACTTTGTACAAGAAAGCTGGGTA*GTCTTGTTTGCGAGAATTGGC*
*OLE1*F	At4g25140	ACAAGTTTGTACAAAAAAGCAGGCTCTCCAACCACC*ATGGCGGATACAGCTAGAGGAAC*
*OLE1*R		TCCGCCACCACCAACCACTTTGTACAAGAAAGCTGGGTA*AGTAGTGTGCTGGCCACC*
*ATG8b*F	At4g04620	ACAAGTTTGTACAAAAAAGCAGGCTCTCCAACCACC*ATGGAGAAGAACTCCTTCAAG*
*ATG8b*R		TCCGCCACCACCAACCACTTTGTACAAGAAAGCTGGGTA*GCAGTAGAAAGATCCACC*
*ATG8h*F	At3g06420	ACAAGTTTGTACAAAAAAGCAGGCTCTCCAACCACC*ATGGGGATTGTTGTCAAGTCT*
*ATG8h*R		TCCGCCACCACCAACCACTTTGTACAAGAAAGCTGGGTA*GCCGAAAGTTTTCTCGGT*
AIM1F	At4g26740	CCGGGGTGGTTACCTTCA
AIM1R		GGTCAGGTTGATAACAGCG
AIM2F	At4g26740	TACTTGCTAGCAAGGGATG
AIM2R		GCCTGCGATCCATCCAAA
NtermF	At4g26740	ACAAGTTTGTACAAAAAAGCAGGCTCTCCAACCACC*ATGATCATTGGGTCGCTTATAATAG*

Supplemental Table S6. The nomenclature of caleosins from Arabidopsis used in the literature.

Locus ID	The nomenclature used in this paper according to Shen et al. (2014)[1]	Alternative nomenclature used in Song et al. (2014)[2]
At4g26740	CLO1	CLO1
At5g55240	CLO2	CLO2
At2g33380	CLO3	CLO3
At1g70670	CLO4	CLO4
At1g23240	CLO5	CLO8
At1g70680	CLO6	CLO5
At1g23250	CLO7	CLO7
At5g29560	CLO8	CLO6

[1] also used in Shimada and Hara-Nishimura (2015); Shen et al. (2016); Hanano et al. (2016) Shao et al. (2019).

[2] also used in de Vries and Ischebeck (2020).

de Vries J, Ischebeck T (2020) Ties between Stress and Lipid Droplets Pre-date Seeds. Trends Plant Sci **25:** 1203-1214

Hanano A, Almousally I, Shaban M, Rahman F, Hassan M, Murphy DJ (2016) Specific Caleosin/Peroxygenase and Lipoxygenase Activities Are Tissue-Differentially Expressed in Date Palm (Phoenix dactylifera L.) Seedlings and Are Further Induced Following Exposure to the Toxin 2,3,7,8-tetrachlorodibenzo-p-dioxin. Front Plant Sci **7:** 2025

Kalvari et al. (2014) iLIR: A web resource for prediction of Atg8-family interacting proteins. Autophagy **10:** 913-925

Poxleitner M, Rogers SW, Lacey Samuels A, Browse J, Rogers JC (2006) A role for caleosin in degradation of oil-body storage lipid during seed germination. Plant J **47:** 917-933

Purkrtova Z, d'Andrea S, Jolivet P, Lipovova P, Kralova B, Kodicek M, Chardot T (2007) Structural properties of caleosin: a MS and CD study. Arch Biochem Biophys **464:** 335-343

Shao Q, Liu X, Su T, Ma C, Wang P (2019) New Insights Into the Role of Seed Oil Body Proteins in Metabolism and Plant Development. Front Plant Sci **10:** 1568

Shen Y, Jia Q-L, Liu M-Z, Li Z-W, Wang L-L, Zhao C-Z, Li Z-X, Zhang M (2016) GENOME-WIDE CHARACTERIZATION AND PHYLOGENETIC AND EXPRESSION ANALYSES OF THE CALEOSIN GENE FAMILY IN SOYBEAN, COMMON BEAN AND BARREL MEDIC. Archives of Biological Sciences **68:** 575-585

Shen Y, Xie J, Liu R-d, Ni X-f, Wang X-h, Li Z-x, Zhang M (2014) Genomic analysis and expression investigation of caleosin gene family in Arabidopsis. Biochemical and Biophysical Research Communications **448:** 365-371

Shimada TL, Hara-Nishimura I (2015) Leaf oil bodies are subcellular factories producing antifungal oxylipins. Curr Opin Plant Biol **25:** 145-150

Song W, Qin Y, Zhu Y, Yin G, Wu N, Li Y, Hu Y (2014) Delineation of plant caleosin residues critical for functional divergence, positive selection and coevolution. BMC Evolutionary Biology **14:** 124
